# In vivo neural regeneration via AAV-NeuroD1 gene delivery to astrocytes in neonatal hypoxic-ischemic brain injury

**DOI:** 10.1186/s41232-024-00349-y

**Published:** 2024-07-16

**Authors:** Miri Kim, Seokmin Oh, Songyeon Kim, Il-Sun Kim, Joowon Kim, Jungho Han, Ji Woong Ahn, Seungsoo Chung, Jae-Hyung Jang, Jeong Eun Shin, Kook In Park

**Affiliations:** 1 Yonsei Biomedical Research Institute, Yonsei University College of Medicine, Seoul, 03722 Republic of Korea; 2 Department of Pediatrics, Severance Children’s Hospital, Yonsei University College of Medicine, 50-1 Yonsei-Ro, Seodaemun-Gu, Seoul, 03722 Republic of Korea; 3 Department of Chemical and Biomolecular Engineering, Yonsei University, Seoul, 03722 Republic of Korea; 4 Department of Physiology, Brain Korea 21 Plus Project for Medical Science, Yonsei University College of Medicine, Seoul, Republic of Korea; 5 BnH Research. Co., Ltd. Goyang-Si, Gyeonggi-Do, Republic of Korea; 6 GluGene Therapeutics Inc., Seoul, Republic of Korea

**Keywords:** In vivo direct reprogramming, Adeno-associated virus, Neurogenic differentiation factor 1, Hypoxic-ischemic brain injury

## Abstract

**Background:**

Neonatal hypoxic-ischemic brain injury (HIBI) is a significant contributor to neonatal mortality and long-term neurodevelopmental disability, characterized by massive neuronal loss and reactive astrogliosis. Current therapeutic approaches for neonatal HIBI have been limited to general supportive therapy because of the lack of methods to compensate for irreversible neuronal loss. This study aimed to establish a feasible regenerative therapy for neonatal HIBI utilizing in vivo direct neuronal reprogramming technology.

**Methods:**

Neonatal HIBI was induced in ICR mice at postnatal day 7 by permanent right common carotid artery occlusion and exposure to hypoxia with 8% oxygen and 92% nitrogen for 90 min. Three days after the injury, NeuroD1 was delivered to reactive astrocytes of the injury site using the astrocyte-tropic adeno-associated viral (AAV) vector AAVShH19. AAVShH19 was engineered with the Cre-FLEX system for long-term tracking of infected cells.

**Results:**

AAVShH19-mediated ectopic NeuroD1 expression effectively converted astrocytes into GABAergic neurons, and the converted cells exhibited electrophysiological properties and synaptic transmitters. Additionally, we found that NeuroD1-mediated in vivo direct neuronal reprogramming protected injured host neurons and altered the host environment, i.e., decreased the numbers of activated microglia, reactive astrocytes, and toxic A1-type astrocytes, and decreased the expression of pro-inflammatory factors. Furthermore, NeuroD1-treated mice exhibited significantly improved motor functions.

**Conclusions:**

This study demonstrates that NeuroD1-mediated in vivo direct neuronal reprogramming technology through AAV gene delivery can be a novel regenerative therapy for neonatal HIBI.

**Supplementary Information:**

The online version contains supplementary material available at 10.1186/s41232-024-00349-y.

## Introduction

Neonatal hypoxic-ischemic brain injury (HIBI) accounts for most neonatal central nervous system injuries and leads to neonatal death and long-term neurodevelopmental deficits, including cognitive and learning disabilities, cerebral palsy, and epilepsy [1]. Despite the introduction of therapeutic hypothermia and advancements in supportive care, optimal therapeutics to improve the neurological outcomes of HIBI patients are still lacking [2]. Of note, the regeneration of functional neurons to replace damaged ones remains an important challenge for clinicians.

In the acute phase of HIBI, astrocytes proliferate and migrate to the lesion sites, forming a barrier for toxic substances. However, once activated, reactive glial cells remain and accumulate at the lesion site, forming a glial scar and secreting neuroinhibitory factors that block axon regeneration [3]. Therefore, the accumulation of reactive astrocytes during the later stages after HIBI is considered an obstacle to regenerative therapies, including both endogenous neuroregeneration or exogenous cell transplantation. Recently, in vivo direct neuronal reprogramming technology has emerged as a promising approach for replenishing lost neurons without the need for exogenous cell supply or immunosuppression [4, 5]. Furthermore, ectopic expression of regulatory transcription factors (e.g., Neurogenin2, Sox2, Ascl1, and neurogenic differentiation factor 1 (NeuroD1)) convert glial cells into functional neurons after brain or spinal cord injury [6–10].

In this study, we established a neuroregenerative strategy for treating neonatal HIBI through astrocyte-to-neuron conversion by ectopic NeuroD1 expression. NeuroD1 was delivered to reactive astrocytes of HIBI mice, using the astrocyte-tropic adeno-associated viral (AAV) vector AAVShH19, engineered with the Cre-FLEX system. We investigated the efficiency of in vivo astrocyte-to-neuron conversion and verified the functional status of neurons in terms of electrophysiological activities and synaptic connections with existing host neurons. Changes in the microenvironment of HIBI and recovery from neurobehavioral deficits were also analyzed.

## Methods

### Primary astrocyte isolation and culture

Primary astrocytes were prepared from the ICR mouse cortex on postnatal day 3 using a previously published protocol [11]. Briefly, meninges were removed, the cortex was carefully dissected, and the tissue was digested with trypsin-ethylene-diamine-tetraacetic acid (trypsin–EDTA; Gibco, Carlsbad, CA, USA) for 30 min at 37 °C. After centrifugation (300 g, 5 min), the cell pellet was suspended in astrocyte culture medium (Dulbecco’s modified Eagle’s medium; Gibco) containing 10% fetal bovine serum (FBS; Gibco) and 0.1% penicillin/streptomycin (Gibco)). These mixed glia cells were plated in a poly-D-lysine-coated T75 tissue culture flask, and the medium was changed to a fresh medium every 2–3 days. After 1 week, microglia were separated from astrocyte monolayer sheets by shaking the flask at 180 rpm for 30 min and then discarding the supernatant. Next, 20 mL of fresh medium was added, the flask was shaken at 240 rpm for 6 h, and the supernatant was discarded to remove oligodendrocytes. The remaining astrocyte layer was rinsed with phosphate-buffered saline (PBS; Promega, Madison, WI, USA). After aspiration of PBS, 5 mL of trypsin–EDTA (Gibco) was added at 37 °C for 10 min, then 5 mL of fresh medium was added, and the cells were spun at 180 g for 5 min. The supernatant was discarded, and 40 mL of fresh medium was added and replaced every 2–3 days. These primary cultured astrocytes were used for neuronal conversion.

### AAV vector transgene construction

To construct pAAVShH19-CMV-NeuroD1-T2A-GFP, the NeuroD1, T2A, and GFP genes were inserted into the pAAVShH19-Multi-cloning site (MCS) (Cell Biolab #VPK-410, San Diego, CA, USA) between EcoRI and HindIII using Gibson assembly cloning kit (New England Biolabs, Ipswich, MA, USA) according to the manufacturer’s protocol. To construct the pAAVShH19-hGFAP-Cre vector, the hGFAP promoter and the Cre gene were obtained from the hGFAP-Cre plasmid (RRID:Addgene_40591, Watertown, MA, USA) and inserted into pAAVShH19-MCS between MluI and EcoRI to replace the CMV promoter and human β-globin intron. To construct pAAVShH19-FLEX-NeuroD1-T2A-GFP and pAAVShH19-GFP-T2A-GFP, pAAVShH19-FLEX-GFP (RRID:Addgene_28304) was used as the backbone. The NeuroD1, T2A, and GFP genes were inserted into the backbone between KpnI and XhoI using the Gibson assembly cloning kit (New England Biolabs) according to the manufacturer’s protocol.

### AAV vector production

To produce AAV vectors, HEK293T cells derived from human embryonic kidney cells (ATCC, Manassas, VA, USA) were cultured in Dulbecco’s modified Eagle’s medium (Corning, Glendale, AZ, USA) supplemented with 10% FBS (Corning) and 1% penicillin/streptomycin (Gibco) at 37 °C under 5% CO_2_. The recombinant AAVshH19 vector, which was engineered to enhance its affinity to glial cells in a previous study [12], was produced in HEK293T cells using a calcium-phosphate transient transfection method as previously described [13]. Equal quantities of three plasmids (17 μg each), including the one with a rep/AAVShH19 cap gene (a kind gift from Dr. David Schaffer, University of California, Berkeley), a transgene, and an adenoviral helper gene (pHelper), were transfected into HEK293T cells by complexing with a calcium-phosphate solution. Cells were harvested 72 h post-transfection and lysed by three cycles of freezing and thawing. AAVShH19 crude lysate was treated with a benzonase nuclease (Sigma, St. Louis, MO, USA) and purified by centrifugation at 42,000 rpm for 2 h in discontinuous iodixanol density gradients (Axis-Shield, Dundee, Scotland, UK) with a VTi 65·2 rotor (Beckman Coulter, Brea, CA, USA). The virus-containing layer was extracted, and the buffer exchange was conducted with PBS (Biosesang, Seongnam, Korea) containing 0.01% (v/v) Tween-20 (Sigma) using 100 K MWCO Amicon tubes (Millipore, Billerica, MA, USA) according to the manufacturer’s instructions. DNase-resistant viral genome was extracted by Proteinase K (both from Thermo Fisher Scientific, Waltham, MA, USA) and then quantified by quantitative PCR (Mini Opticon; Bio-Rad, Hercules, CA, USA) using the SYBR Green master mix (Thermo Fisher Scientific).

### Analysis of astrocyte-to-neuron conversion in vitro

Primary astrocytes (1.5 × 10^4^ cells/100 μL) were plated in eight-well plates and infected with AAVShH19-CMV-GFP (1 × 10^9^ vg) or AAVShH19-CMV-NeuroD1-GFP (1 × 10^9^ vg). After 24 h, the culture medium was completely replaced with differentiation medium containing neurobasal medium (Gibco), 0.5% FBS (Gibco), GlutaMAX (Sigma), penicillin/streptomycin (Gibco), B27 (Gibco), and N2 supplement (Gibco). Infected cells were cultured for 14 or 30 days, and their reprogramming patterns were examined by immunocytochemical analysis. Cells were fixed with 4% paraformaldehyde for 20 min at room temperature. Next, they were blocked with a solution containing 0.3% Triton X-100, 3% bovine serum albumin (Sigma), and 10% normal donkey serum (Jackson ImmunoResearch, West Grove, PA, USA) in PBS for 1 h. The cells were then incubated with the following primary antibodies overnight at 4 °C: rabbit anti-glial fibrillary acidic protein (GFAP; 1:1000; Dako, Cat #Z0334); mouse anti-NeuN (1:40; Chemicon, Cat #MAB377); rabbit anti-ionized calcium-binding adapter molecule 1 (Iba-1; 1:500; Wako, Cat #019–19741); mouse anti-neuronal class III β-tubulin (TUJ1; 1:500; Covance, Cat #MMS-435P); goat anti-choline acetyltransferase (ChAT; 1:200; Millipore, Cat #AB144P); guinea pig anti-vGLUT1 (1:1500; Chemicon, Cat #AB5905); rabbit anti-NeuN (1:500; Millipore, Cat #ABN78); mouse anti-GABA (1:200; Sigma, Cat #A0310); mouse anti-GAD67 (1:1000; Chemicon, Cat #MAB5406); rabbit anti-MAP2 (1:300; Cell Signaling Technology, Inc., Cat #4542); and mouse anti-s100b (1:1500; Sigma, Cat #S2532). After washing three times with PBS, the cells were further incubated with Alexa Fluor secondary antibodies (1:500; Life Technologies, Carlsbad, CA, USA) for 70 min at 37 °C in darkness, followed by three PBS washes. Nuclei were counterstained using 40,6-diamidino-2-phenylindole (DAPI), and then each sample was mounted onto glass slides using a Vectashield mounting medium (Vector, Burlingame, CA, USA). Immunofluorescence images were acquired by immunofluorescence microscopy (BX51; Olympus, Center Valley, PA, USA).

### Induction of HIBI and virus injection

The Institutional Animal Care and Use Committee at Yonsei University College of Medicine, Seoul, Korea (permit number: 2019–0011) approved all procedures, which were conducted in accordance with the guidelines of the National Institutes of Health. On postnatal day 7, ICR mice were anesthetized with isoflurane and underwent permanent occlusion of the right common carotid artery. Following the procedure, the pups were kept warm until regaining consciousness and were returned to their dams for 2 h. After this, the pups were exposed to a hypoxic atmosphere consisting of 8% oxygen and 92% nitrogen for a duration of 1.5 h. The body temperature of the mice was maintained at 37 °C using a warm pad. All mice received the same housing and care, including a 12:12 h light-to-dark cycle, and were evenly distributed among the vehicle, control, and NeuroD1 groups. On day 3 after inducing HIBI, the mice were randomly divided into three groups for AAVShH19 or vehicle injection: (i) PBS (vehicle, 6 µL); (ii) AAV-GFAP::Cre (1.5 × 10^10^ vg/µL) together with AAV-CAG::FLEX-GFP-T2A-GFP (1.5 × 10^9^ vg/µL; control AAV-GFP, 6 µL); (iii) AAV-GFAP::Cre (1.5 × 10^10^ vg/µL) together with AAV-CAG::FLEX-NeuroD1-T2A-GFP (1.5 × 10^9^ vg/µL; AAV-NeuroD1-GFP, 6 µL). The mice were anesthetized and then dissected through the dorsal midline of the scalp. A glass micropipette (0.3 mm in diameter) was used to inject AAV-GFAP::Cre + AAV-CAG::FLEX-GFP, AAV-GFAP::Cre + AAV-CAG::FLEX-NeuroD1-T2A-GFP, or an equivalent amount of vehicle directly into the center of the infarct site. The investigator was blinded to the group allocation of the animals during the experiment. No statistical method was used to predetermine the sample size. The sample size of each experiment is shown in the figure legends. No data were excluded from the analysis.

### Immunohistochemistry and analysis

For brain slice immunohistochemical analysis, mice were deeply anesthetized and intracardially perfused with ice-cold PBS and then 4% paraformaldehyde in PBS. The brains were surgically dissected, post-fixed in 4% paraformaldehyde at 4 °C overnight, cryoprotected with 30% sucrose solution at 4 °C for 48 h, and frozen in optimal cutting temperature compound (Sakura Finetek, Torrance, CA, USA). Frozen brains were coronally cut into 16-μm sections by a cryomicrotome (Cryostat Leica, Wetzlar, Germany). Brain sections were permeabilized two times in PBS for 3 min each and blocked in PBS containing 10% normal donkey serum, 0.3% Triton X-100, and 3% bovine serum albumin for 1 h at room temperature. Sections were then incubated overnight at 4 °C in a humid environment with the following primary antibodies: rabbit anti-Iba-1 (1:500; Wako); mouse anti-synaptophysin (1:200; Sigma, Cat #S5768); mouse anti-SMI311 (1:250; Covance, Cat #SMI-311R); guinea pig anti-GFP (1:500; Cosmo bio, Cat #GP-Af1180); rabbit anti-Olig2 (1:500; Millipore, Cat #AB9610); goat anti-doublecortin (1:200; Santa Cruz, Cat #SC8066); mouse anti-nestin (1:100; Chemicon, Cat #MAB353); mouse anti-NeuN (1:40; Chemicon); rabbit anti-GFAP (1:1500; Dako); rabbit anti-TUJ1 (1:500; Covance); goat anti-ChAT (1:200; Millipore); guinea pig anti-vGLUT1 (1:1500; Chemicon); rabbit anti-NeuN (1:500; Millipore); rabbit anti-VGAT (1:50; Cell signaling, Cat#44,498); rabbit anti-GABA (1:1000; Sigma, Cat #A2052); mouse anti-GAD67 (1:1000; Chemicon); rabbit anti-MAP2 (1:300; Cell Signaling Technology, Inc.); mouse anti-S100β (1:1500; sigma); rabbit anti-glutamine synthetase (1:250; Sigma, Cat #G2781); and rabbit anti-NG2 (1:200; Chemicon, Cat #AB5320). After washing three times with PBS, the sections were incubated for 70 min at 37 °C in darkness with appropriate secondary antibodies conjugated to Alexa Fluor 405, 488, or 594 (1:400; Life Technologies) containing 3% normal donkey serum. This was followed by three PBS washes, and nuclei were counterstained with DAPI. Finally, immunofluorescence images were captured using a BX51 or confocal laser scanning microscope (LSM 700; Carl Zeiss, Oberkochen, Germany). Positive cells in five random fields were counted. For quantification, each field was randomly scanned at 200 × and 400 × magnifications. In three to six random fields, positive cells were counted in the area of viral infection proximal to the infarction. Image collector and analysis were performed blindly.

### RNA extraction from brain tissues of the HIBI model

Total RNA was extracted from tissue dissected from the ipsilateral hemisphere with TRIzol reagent (Molecular Research Center, Cincinnati, OH, USA) according to the manufacturer’s instructions. The purity of the RNA was evaluated by the A260/A280 ratio, and the RNA concentration was measured using an Agilent 2100 Bioanalyzer (Agilent Technologies, Palo Alto, CA, USA). The extracted RNA samples were divided into aliquots and stored at − 80 °C until further use.

### Quantitative real-time polymerase chain reaction (qRT-PCR)

To perform gene expression analysis, first-strand cDNA was synthesized using 1 μg of total RNA and a first-strand cDNA synthesis kit (Promega) following the manufacturer’s protocol. The cDNA was then amplified using a LightCycler 480 SYBR Green I Master mix (Roche, Mannheim, Germany) in a LightCycler 480 System real-time qPCR machine (Roche). The internal control gene used was Gapdh. The amplification product’s specificity was confirmed by melting curve analysis. The following primer pairs were used, with the sense primer listed first followed by the antisense primer: (1) TNF-α: 5′- CCAGTGTGGGAAGCTGTCTT -3′ and 5′-AAGCAAAAGAGGAGGCAACA-3′; (2) IL1β: 5′-TTCAGGCAGGCAGTATCACTC-3′ and 5′-GAAGGTCCACGGGAAAGACAC-3′; (3) IL6: 5′-TAGTCCTTCCTACCCCAATTTCC-3′ and 5′-TTGGTCCTTAGCCACTCCTTC-3′; (4) iNOS: GGAGTGACGGCAAACATGACT and 5′-TAGCCAGCGTACCGGATGA-3′ (5) GAPDH: 5′-AGGACCAGGTTGTCTCCTGC-3′ and 5′-ACCCTGTTGCTGTAGCCGT-3′; (6) GBP2: 5′-AATTGAGAGTGAGGCCATTG-3′ and 5′-AGCTGATGAGACATCCATGT-3′; (7) LCN2: 5′-AGGCAGCTTTACGATGTACA-3′ and 5′-CTGGAGCTTGGAACAAATGT-3′; (8) SERPING1: 5′-TGACCATACTTTGAAGGCCA-3′ and 5′-ATGATGGCCTTGAAGACAGT-3′; (9) C3: 5′-GAGGAAGCCAAGAATACCAT-3′ and 5′-TATCTATCTACTCCAGAGGC-3′; (10) SYN1: 5′-CTGCTGAGCCCTTCATTGATG-3′ and 5′-TGGTCTTCCAGTTACCCGACA-3′; (11) GFAP: 5′-AATGCTGGCTTCAAGGAGAC-3′ and 5′-CCTTGTTTTGCTGTTCCAGG-3′; (12) NEUN: 5′-CAGCCATTGGCATATGGGTT-3′ and 5′-AAGCTGAATGGGACGATCGT-3′; (13) MAP2: 5′-AAAGAGAACGGGATCAACGG-3′ and 5′-TTCAGGACTGCTACAGCTTC-3′; (14) TUJ1: 5′-CCATTCTGGTGGACTTGGAA-3′ and 5′-GCACCACTCTGACCAAAGAT-3′.

### Electrophysiological analysis

At 60 dpi, mice were anesthetized with 5% isoflurane and perfused transcardially with ice-cold sucrose-based artificial cerebrospinal fluid (aCSF), which contained (in mM) 195.5 sucrose, 32.5 NaHCO_3_, 11 glucose, 2.5 KCl, 2 Na pyruvate, 1 NaH_2_PO_4_, 1 Na L-ascorbate, 5 MgSO_4_, and 0.5 CaCl_2_ saturated with 95% O_2_–5% CO_2_ to pH 7.3–7.4. The brain was quickly removed and placed into the slicing chamber containing the same ice-cold sucrose-based aCSF. Four hundred-micrometer thick coronal brain slices were obtained. The slices were incubated in aCSF at 35 °C for 30 min for recovery and moved to aCSF solution at room temperature for 1–4 h before being placed in the recording chamber for the experiment. The standard aCSF contained (in mM) 124 NaCl, 26.2 NaHCO_3_, 11 glucose, 2.5 KCl, 2.5 CaCl_2_, 1.3 MgSO_4_, 1 NaH_2_PO_4_, 2 Na pyruvate, and 1 Na L-ascorbate saturated with 95% O_2_–5% CO_2_ to pH 7.3–7.4. All electrophysiological recordings were performed at 23–25 °C.

Whole-cell patch-clamp recordings were obtained from healthy-appearing GFP + cells of the ipsilateral hemisphere. Recording pipettes with 3–6 MΩ tip resistance was made using a micropipette puller (Narishige, PC-100, Amityville, NY, USA) and filled with internal solution containing (in mM) 135 K-gluconate, 10 HEPES, 8 NaCl, 5 Phosphocreatine di (Tris), 4 Mg-ATP, 0.4 Na_2_GTP, and 0.5 EGTA, pH 7.35 with KOH and 285 mOsm. Black-and-white images of the recorded neuron under infrared and fluorescence illumination were captured (HCI live Image, Hamamatsu Photonics, Shizuoka, Japan). Recording cells were clamped with 500-ms current pulses from − 100 to 350 pA amplitude with 50 pA increments, and corresponding voltage responses were recorded with a 100 kHz sampling rate (pCLAMP 11, Axon Instruments, San Jose, CA, USA).

### Behavioral tests and analysis/behavioral assessment

#### Adhesive removal test

The adhesive removal test was performed to evaluate somatosensory and motor functions and deficits according to a previously described procedure [14]. A mouse was placed in a test box (150 cm^2^) for 1 min to habituate. Thereafter, the mouse was taken out of the box, and two small pieces of adhesive tape (0.3 × 0.3 cm) were attached with equal pressure to each forepaw. The order of tape application (right or left) was alternated between each animal. The mouse was then placed in a test box, and the time to first contact and removal from each paw was recorded. A test time of up to 120 s was allowed for mice to remove the adhesive tape. The adhesive removal test was performed 2, 3, and 4 weeks after virus injection.

#### Rotarod test

To evaluate motor coordination and balance, the rotarod test was conducted using a knurled white plastic rod with a diameter of 3 cm and a width of 6 cm (LSi Letica; Panlab, Barcelona, Spain). The mice were placed on the rotating rod (4 rpm) in the opposite direction of their movement to become familiar with the setup. The rotational speed was gradually increased up to 40 rpm and then maintained at this final speed for 5 min; the latency to fall from the rod was recorded. The experiment was terminated when the animal fell off the rung or grabbed the apparatus and rotated twice in succession. The average of three consecutive trials was considered as the latency to fall off the rod. A researcher who was blinded to the treatment groups randomized all neonatal HI-injured mice into three experimental groups (vehicle, control, and NeuroD1).

### Statistics

All cell experiments were performed in triplicate. No statistical methods were used to predetermine the sample size. The Statistical Package for Social Sciences (SPSS) software (version 25; IBM Corporation, Armonk, NY, USA) was used for statistical analysis. Data are represented as mean ± standard error of the mean (SEM). One-way analysis of variance was used to compare multiple groups, followed by Bonferroni post hoc analysis for pairwise comparisons of groups. A Student’s *t* test (two-tailed unpaired) was used when comparing two values. *p* < 0.05 indicated a statistically significant difference.

## Result

### NeuroD1 converts cultured astrocytes into functional neurons

**Fig. 1:**
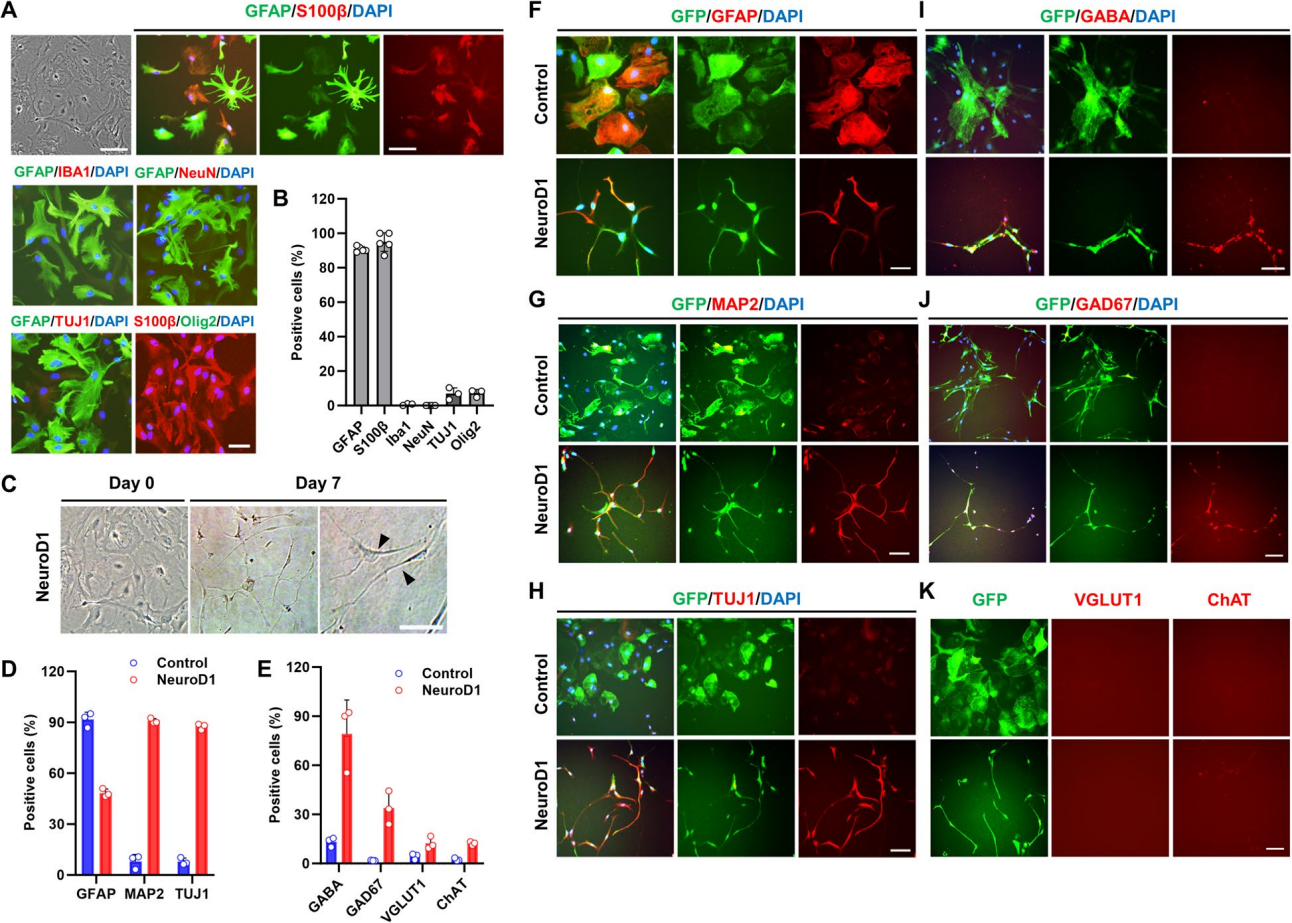
HYPERLINK "sps:id::fig1||locator::gr1||MediaObject::0" The expression of NeuroD1 in cultured astrocytes induced neuronal differentiation. **A** The cultured cells were positive for the markers of astrocytes (GFAP and S100β) but negative for markers of neurons (NeuN and TUJ1), oligodendrocytes (Olig2), and microglia (Iba1). Scale bar, 50 µm. **B** Quantification analysis of GFP + cells co-labeled with each marker (*n* = 3–5). Data are presented as means ± SEM. **C** Morphological transition from astrocytes (left) to neurons (right, arrowhead) by AAVShH19-CMV-NeuroD1-T2A-GFP infection (NeuroD1). Representative images on day 7 are shown. Scale bar, 50 µm. **D**, **F**–**H** Representative images and percentage quantification of astrocytic (GFAP) and neuronal markers (MAP2 and TUJ1) in primary astrocytes cultured in neuronal differentiation medium for 2 weeks after infection with AAVShH19-CMV-NeuroD1-T2A-GFP (NeuroD1) or AAVShH19-CMV-GFP (control). Scale bar, 50 µm (*n* = 3). Data are presented as means ± SEM. **E**, **I**, **J** Representative images and percentage quantification of GABAergic neuronal markers (GABA and GAD67). **K** The newly converted neurons did not express glutamatergic neuronal (VGLUT1) or cholinergic neuronal markers (ChAT). Scale bar, 50 µm (*n* = 3). Data are presented as means ± SEM

To investigate whether NeuroD1 could convert astrocytes into functional neurons, we cultured primary astrocytes obtained from the early postnatal mouse cerebral cortex (Fig. 1A). Most cultured mouse astrocytes expressed the astrocytic markers glial fibrillary acidic protein (GFAP, 90.8 ± 0.7%) and S100 calcium-binding protein beta (S100β, 95.0 ± 2.4%). Very few cells expressed markers for microglia (anti-ionized calcium-binding adapter molecule 1 (Iba1 +), 0.6 ± 0.3%), neurons (neuronal nuclei (NeuN +), 0.0 ± 0.0%; neuronal class III β-tubulin (Tuj1 +), 6.9 ± 2.0%), or oligodendrocyte precursors (Oligodendrocyte transcription factor 2 (Olig2 +), 7.2 ± 1.3%) (Fig. 1B). These results indicate that the cultured cells were indeed astrocytes and not neurons, microglia, or oligodendrocytes. Based on a previous study [12], AAVShH19 (an AAV2-like variant with highly efficient astrocyte transduction) was used as a viral vector in this study. NeuroD1 and green fluorescence protein (GFP) were driven by the cytomegalovirus (CMV) promoter, and transduced cells expressing the NeuroD1 gene were easily monitored by inserting a T2A self-cleaving peptide sequence between the NeuroD1 and GFP genes (AAV-CMV-NeuroD1-T2A-GFP; Additional file 1: Fig. S1). We first investigated the potential of NeuroD1 for astrocyte-to-neuron conversion by infecting cultured mouse astrocytes with either AAV-CMV-NeuroD1-T2A-GFP or AAV-CMV-GFP (control). The next day after infection, the medium was switched to neuronal induction medium, and NeuroD1-infected cells exhibited bipolar processes on day 7 post-infection (Fig. 1C). On day14 post-infection, immunostaining with astrocytic (GFAP) or neuronal (MAP2 and TUJ1) markers showed that NeuroD1-infected cells displayed decreased GFAP signal (91.6 ± 2.5% → 48.3 ± 1.4%) and exhibited neuronal morphology in both GFAP + and GFAP − cells (Fig. 1D, F). Additionally, unlike in the control GFP group, most NeuroD1-infected cells were immunopositive for MAP2 (8.0 ± 2.4% → 90.8 ± 0.9%) and Tuj1 (7.9 ± 1.2% → 87.5 ± 1.0%) (Fig. 1D, G, H). These findings indicate efficient reprogramming of astrocytes into neurons through NeuroD1 expression.

Next, to investigate which types of neurons were differentiated from astrocytes, we stained for Glutamic Acid Decarboxylase 67 (GAD67) and Gamma-aminobutyric acid (GABA; GABAergic neurons); Vesicular glutamate transporter 1 (VGluT1; glutamatergic neurons); and Choline acetyltransferase (ChAT; cholinergic neurons). NeuroD1-infected astrocytes were immunopositive for GAD67 and GABA but not VGluT1 or ChAT (Fig. 1E, I–K). These results demonstrate that NeuroD1 can directly induce the conversion of mouse astrocytes into predominantly GABAergic neurons.

### NeuroD1 reprograms reactive astrocytes into neurons in HIBI

**Fig. 2:**
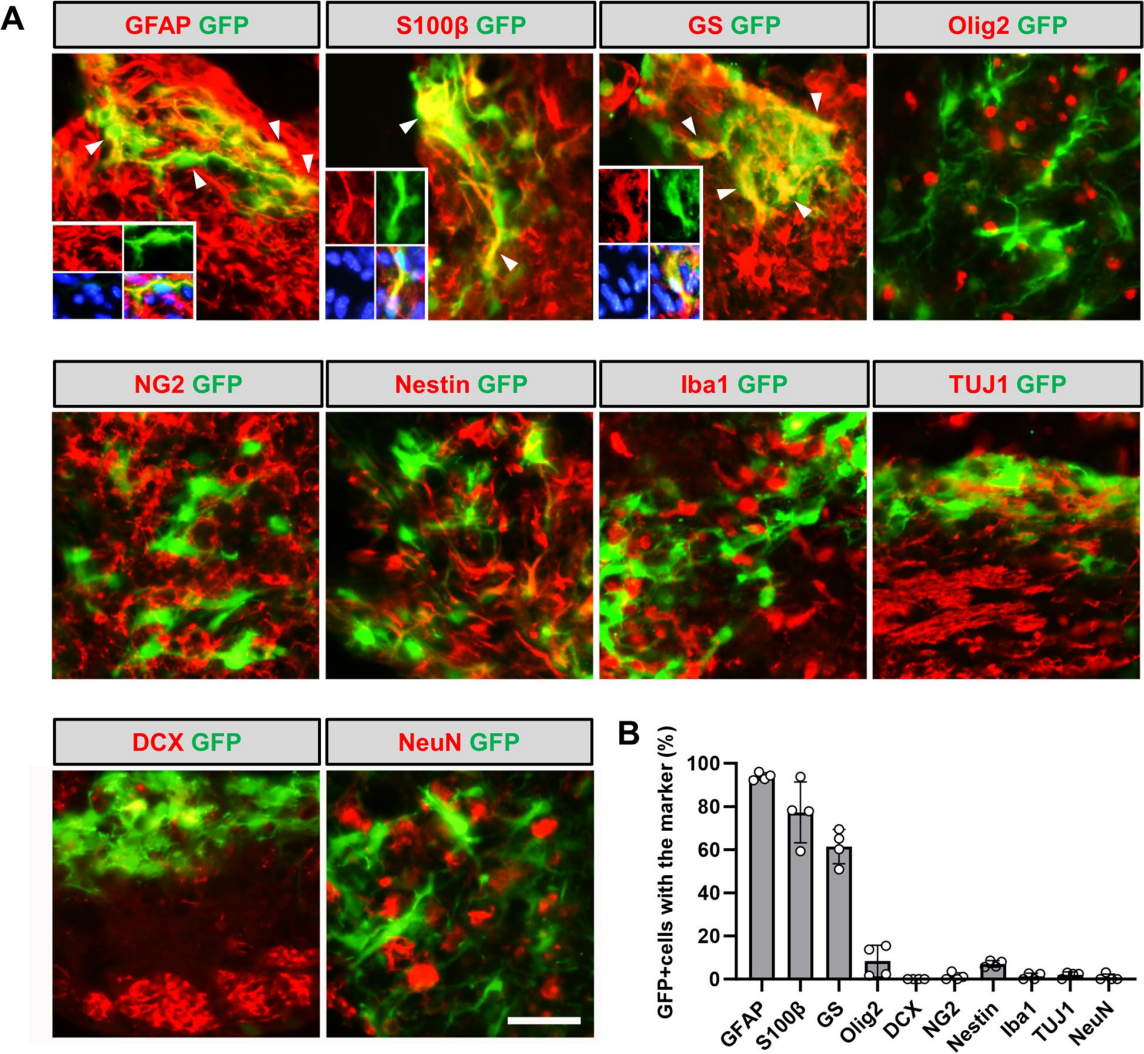
Cell types infected by the AAVShH19 Cre-FLEX system. **A** Representative images showing GFP + cells co-stained with markers for astrocytes (GFAP, S100β, and glutamine synthetase); neurons (NeuN, TUJ1, and doublecortin); microglia (IbaI); oligodendrocytes (Olig2); NG2 glia (NG2); and neural progenitor cells (Nestin). Arrowheads and inset images showing astrocytes co-localized with GFP. Scale bar, 50 µm. **B** Quantitative analysis of GFP + cells co-labeled with each marker in HI-injured brain (*n* = 4 mice). Data are presented as means ± SEM

Brain injury results in neuronal loss and increased activation and proliferation of reactive astrocytes in the affected area [3, 15]. To investigate whether these reactive astrocytes could be converted into functional neurons, AAV encoding NeuroD1 was injected into an HIBI mouse model. To track the transformed neurons from astrocytes long-term, we employed the Cre-FLEX system. This involved simultaneous injection of two vectors: one encoding astrocyte-targeting promoter GFAP-driven Cre recombinase (GFAP::Cre) and the FLEX vector containing double loxP-type recombination sites flanking the reverse sequence of GFP-T2A-GFP or NeuroD1-T2A-GFP under the control of the CAG promoter (CAG::FLEX GFP-T2A-GFP or CAG::FLEX NeuroD1-T2A-GFP) (Additional file 2: Fig. S2). First, AAV-GFAP::Cre with AAV-CAG::FLEX-GFP-T2A-GFP were injected together into the HI injured brain, to confirm the specificity of the Cre-FLEX system. At 3 days post-infection (dpi), most GFP-positive cells expressed the astrocyte-specific markers GFAP (93.9 ± 0.9%), S100β (77.2 ± 4.8%), and glutamine synthetase (62.0 ± 2.4%) but poorly expressed other markers, including Olig2 (9.1 ± 1.8%), NG2 (1.3 ± 0.8%), IbaI (1.3 ± 0.6%), neuroprogenitor marker Nestin (6.9 ± 0.8%), and the neuronal markers doublecortin (0.0 ± 0.0%), TUJ1 (2.0 ± 0.9%), and NeuN (0.8 ± 0.8%) (Fig. 2A, B).

**Fig. 3:**
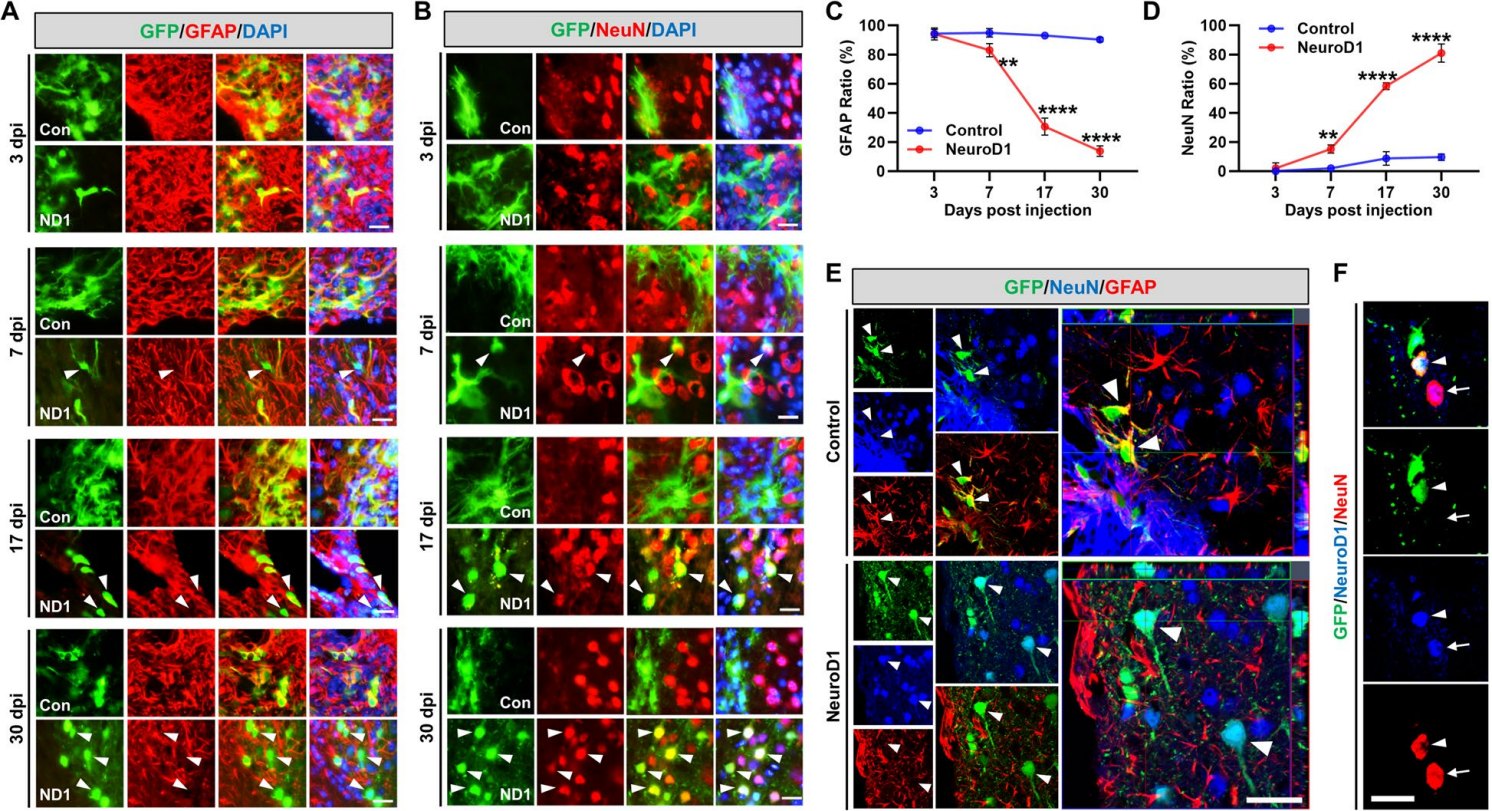
NeuroD1 efficiently converted reactive astrocytes into neurons after hypoxic-ischemic brain injury. **A** Immunostaining of GFP + cells co-stained with GFAP at 3, 7, 17, or 30 dpi in the AAV-GFP (control, top row) and AAV-NeuroD1 (NeuroD1, bottom row) group. Arrowheads indicate loss of GFAP by NeuroD1-mediated conversion. Scale bar, 50 µm. **B** Immunostaining of GFP + cells co-stained with NeuN at 3, 7, 17, or 30 dpi. Arrowheads indicate neurons converted by NeuroD1. Scale bar, 50 µm. **C**, **D** Quantitative analysis of GFP + cells co-labeled with GFAP or NeuN (*n* = 3–4). Data are presented as means ± SEM. **p < 0.01, ***p < 0.001, ****p < 0.0001 vs control. Two-tailed Student’s *t* test. **E** Representative confocal z-stack images showing cells expressing GFP (green, infected cells); GFAP (red, astrocytes); and NeuN (blue, mature neurons). A magnified image of astrocytes merged with GFP (yellow, arrowhead) in the control group and newly converted neurons merged with GFP (light blue, arrowhead) in the NeuroD1 group. Converted cells showed neuronal morphology of an expanded axon. Scale bar, 50 µm. **F** Confocal image showing a converted neuron simultaneously expressing NeuroD1 (blue); GFP (green, infected cells); and NeuN (red, mature neuron). Co-localization of NeuroD1 protein in AAV-NeuroD1-infected cells (GFP + and NeuN + , arrowhead) or preexisting host neurons (NeuN + , arrow). Scale bar, 25 µm

Next, to examine the potential of NeuroD1 in converting astrocytes into neurons, we injected AAV GFAP::Cre together with AAV CAG::FLEX-GFP-T2A-GFP (control AAV-GFP) or AAV CAG::FLEX-NeuroD1-T2A-GFP (AAV-NeuroD1) into HIBI mice. At 3 dpi, both AAV-NeuroD1 and AAV-GFP-infected cells were mainly GFAP + astrocytes (94.7 ± 1.6% and 94.4 ± 1.4%, respectively) with minimal NeuN signal (1.8 ± 1.8% and 0.0 ± 0.0%, respectively). However, at 7 dpi, some AAV-NeuroD1-infected cells started expressing NeuN (15.4 ± 3.1%) and showed significantly decreased GFAP signal (83.1 ± 3.4%) compared to the GFAP signal at 3 dpi, whereas most AAV-GFP-infected cells co-localized with GFAP + astrocytes (94.9 ± 1.5%) and not NeuN (2.1 ± 0.9%) (Fig. 3A–D). At 17 dpi, most AAV-GFP-infected cells remained GFAP + astrocytes (93.2 ± 0.7% GFAP + and only 9.8 ± 2.0% NeuN +), whereas more than half of the AAV-NeuroD1-infected cells lost GFAP (30.6 ± 4.7% GFAP +) and acquired a neuronal identity (56.1 ± 4.1% NeuN +). At 30 dpi, most AAV-NeuroD1-infected cells expressed NeuN (81.0 ± 2.4%), whereas only a small portion retained GFAP (14.0 ± 1.4%). Conversely, the AAV-GFP-infected cells still co-localized with GFAP (90.1 ± 1.0%) and displayed little NeuN signal (9.8 ± 1.1%) (Fig. 3A–D). Quantitative analysis showed that regarding AAV-NeuroD1-infected cells during 3–30 dpi, the number of GFP + cells co-localized with NeuN continuously increased from 1.8 to 81.0%, whereas the number of GFP + cells co-localized with GFAP decreased from 94.7 to 14.0% (Fig. 3C, D). By contrast, AAV-GFP-infected cells predominantly co-expressed GFP and GFAP, with limited NeuN expression throughout the time points. High-magnification confocal images confirmed GFP and GFAP co-expression in AAV-GFP-infected cells, whereas AAV-NeuroD1-infected cells showed co-expression of GFP and NeuN (Fig. 3E). Co-expression of NeuroD1 in converted and non-converted neurons is shown in Fig. 3F (GFP + and NeuN + ; arrowhead, NeuN + ; arrow, respectively).

### Astrocyte-to-neuron conversion by NeuroD1 increases neuronal density in the HI brain

**Fig. 4:**
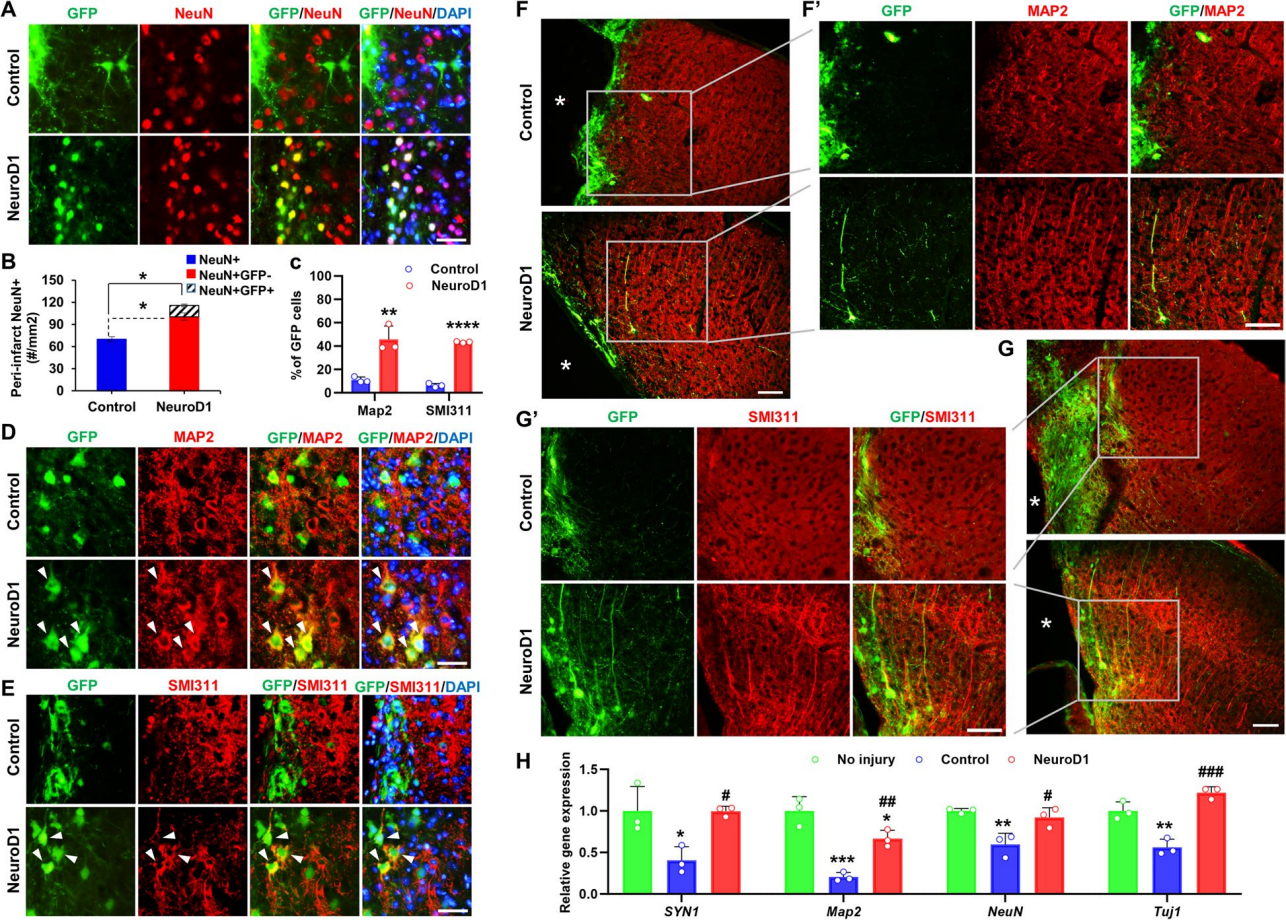
Neuronal recovery in hypoxic-ischemic brain injury after AAV-NeuroD1 injection. **A** Newly converted neurons (yellow) were mixed with preexisting host neurons (red) in the injured site at 30 dpi. Scale bar, 25 µm. **B** Quantitative analysis of total NeuN + cells in the lesion sites of the control AAV-GFP (control) and AAV-NeuroD1 group (NeuroD1) (*n* = 4 mice per group). Data are presented as means ± SEM. *p < 0.05, **p < 0.001 vs control. Two-tailed Student’s *t* test. **C** Quantitative analysis of GFP + cells co-labeled with MAP2 or SMI311 (*n* = 3). Data are presented as means ± SEM. **p < 0.01 and ****p < 0.0001 vs control. Two-tailed Student’s *t* test. **D**, **E** Fluorescence images of neuronal dendritic markers MAP2 (**D**) and SMI311 (**E**) co-localized with NeuroD1-GFP signals (arrowhead). Scale bar, 25 µm. **F** (F’), **G** (G’) Immunostaining of MAP2 (**F**, (F’)) and SMI311 (**G**, (G’)) showed improved neuronal morphology and strong and clearly organized dendrite patterns in the AAV-NeuroD1 group (NeuroD1, bottom) compared with that in the control AAV-GFP group (control, top). The framed areas in f and g are shown at higher magnifications in (F’) and (G’), respectively. An asterisk marks the infarct area. Scale bar, 50 µm. **H** Analysis of neuronal mRNA (Syn1, Map2, Neun, and Tuj1) in the ipsilateral hemisphere of hypoxic-ischemic-injured brain (*n* = 3 mice per group). Data are presented as means ± SEM. *p < 0.05, **p < 0.01, ***p < 0.001 vs no injury; #*p* < 0.05, ##*p* < 0.01, ###*p* < 0.001 vs control. One-way ANOVA followed by a Bonferroni post hoc test

We investigated the effect of astrocyte-converted neurons on neuronal density in the peri-infarct area of HIBI. At 30 dpi, newly-converted neurons (GFP + /NeuN +) and preexisting host neurons (GFP − /NeuN +) coexisted in the injured area, whereas no NeuN + cells were co-localized with GFP in the AAV-GFP group (Fig. 4A). Both total neuronal count (GFP + /NeuN + and GFP − /NeuN + :116 ± 6.8/0.1 mm^2^) and host neuron count (GFP − /NeuN + :100.5 ± 5.7/0.1 mm^2^) of AAV-NeuroD1 group was significantly higher than total neuronal count (NeuN +) of AAV-GFP group (69.8 ± 7.8/0.1 mm^2^, Fig. 4B). NeuroD1-mediated increase in NeuN + neurons was accompanied by increased expression of neurofilament marker SMI311 and neuronal dendritic marker MAP2 in the AAV-NeuroD1 group compared with the AAV-GFP group. Co-localization of NeuroD1 signals with dendrites expressing MAP2 or SMI311 was observed (Fig. 4D, E). In quantitative analysis, the AAV-NeuroD1 group revealed higher percentages of dendrites labeled with MAP2 (45.6 ± 5.4% vs. 11.2 ± 2.5%) and SMI311 (37.9 ± 4.6% vs. 6.3 ± 1.5%) than the AAV-GFP group (Fig. 4C). Furthermore, in the peri-infarct region, the AAV-NeuroD1 group displayed well-preserved and organized dendritic patterns compared with the AAV-GFP group, in which dendrites appeared unaligned with weaker signals (Fig. 4F (F’), G (G’)). qRT-PCR analysis of ipsilateral damaged cortex lysates showed significantly decreased expressions of neuronal genes (Syn1, Map2, Neun, and Tuj1) after HIBI, which were partially restored by NeuroD1-treatment (Fig. 4H). These findings suggest that AAV-NeuroD1-induced astrocyte-to-neuron conversion not only provides protection to damaged neurons but also generates new neurons after HIBI, potentially influencing neuronal recovery.

### Most newly converted neurons are GABAergic neurons and physiologically functional

**Fig. 5:**
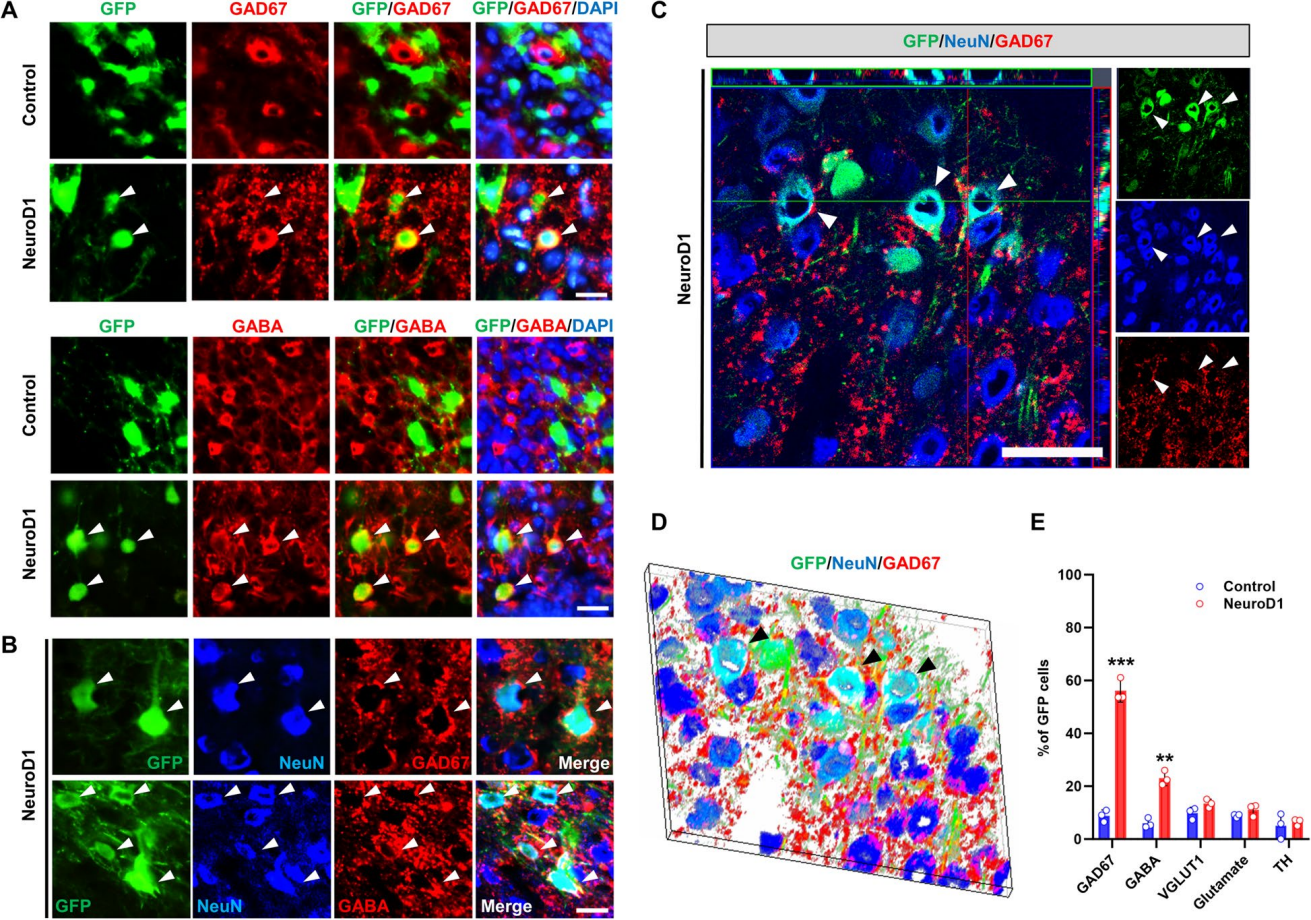
NeuroD1 converted astrocytes into GABAergic neurons in the hypoxic-ischemic-injured brain. **A** Expression of the GABAergic neuronal marker GAD67 or GABA (red) in GFP + cells (green; overlay color: yellow/orange) 4 weeks after NeuroD1 transduction. Scale bar, 25 µm. **B** Expression of the GABAergic neuronal markers GAD67 or GABA (red) in converted neurons showing co-expression of GFP + (green) and NeuN + (blue) 6 weeks after NeuroD1 transduction. Scale bar, 25 µm. **C**, **D** A high-magnification confocal image and the three-dimensional reconstruction of the image showing a converted neuron co-stained with GFP, NeuN, and GAD67. Arrowheads indicate co-labeled cells. Scale bar, 50 µm. **E** Quantitative analysis of GFP + cells co-labeled with GAD67 or GABA. Note that 55.7% and 21.7% of converted cells express GAD67 and GABA, respectively (*n* = 3 mice per group). Data are presented as means ± SEM. **p < 0.01, ***p < 0.001 vs control

We identified the subtypes of newly converted neurons by immunostaining with the neuronal subtype markers GAD67, GABA, VGLUT1, glutamate, and tyrosine hydroxylase for dopaminergic neurons (Fig. 5A–D). At 30 dpi, most GFP-infected cells were co-localized with GAD67^+^ (55.7%) or GABA^+^ (22.8%) in the AAV-NeuroD1 group (Fig. 5A, E) and 48.6% of GFP-infected cells expressed VGAT (Additional file 3: Fig. S3). Triple immunofluorescence for GFP, NeuN, and either GAD67 or GABA revealed the presence of GAD67 or GABA in NeuroD1-converted neurons (Fig. 5B). High-magnification confocal and three-dimensional images at 60 dpi displayed converted neurons with the overlaid markers GFP, NeuN, and GAD67 (Fig. 5C, D).

**Fig. 6:**
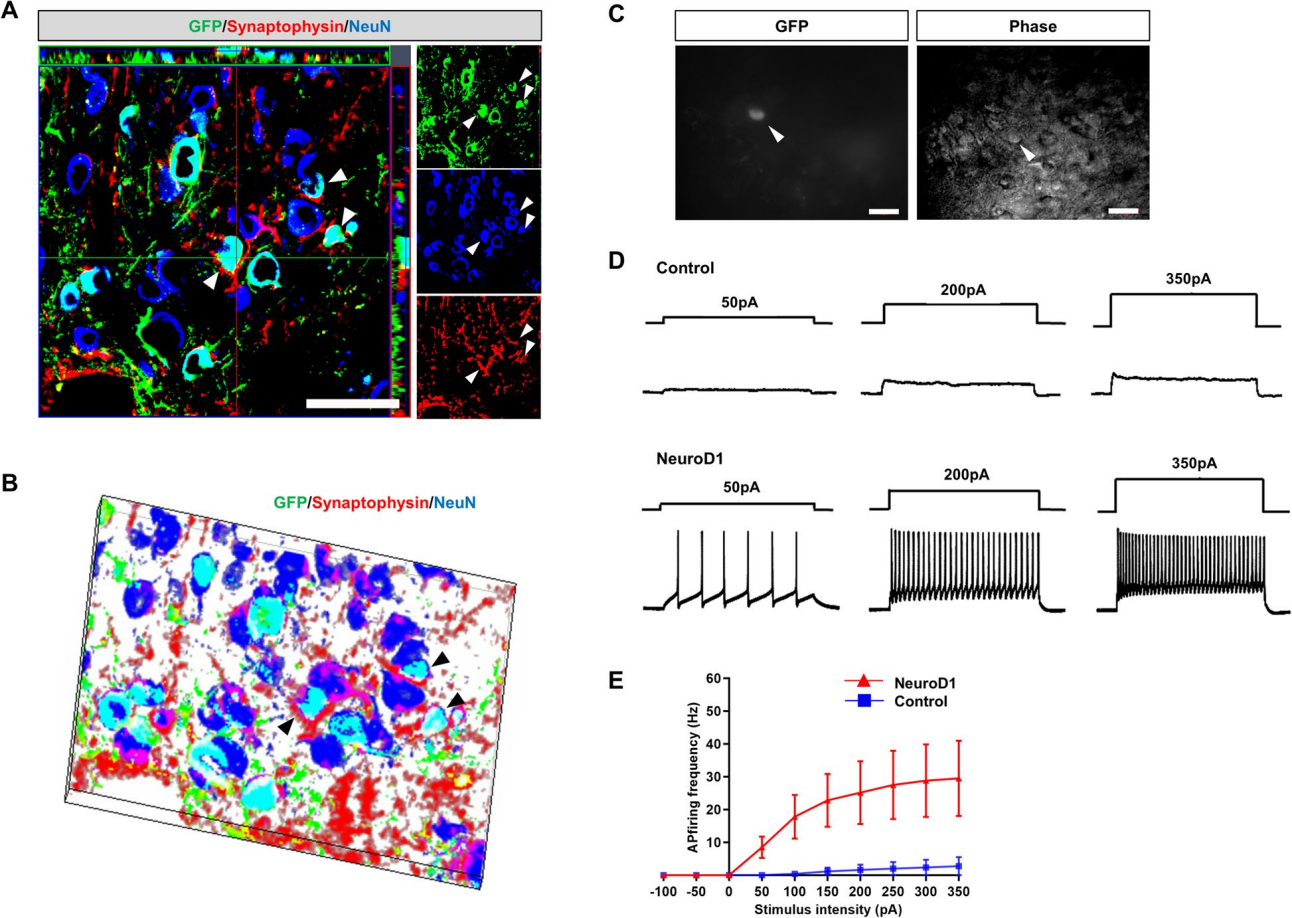
Neurons converted by NeuroD1 were physiologically functional. **A**, **B** Synaptic marker (synaptophysin, red) expression on the newly converted neurons (GFP + and NeuN + , overlay color: light blue) in AAV-NeuroD1-infected cells (green, 60 dpi). A high-magnification confocal image and the three-dimensional reconstruction of the image showing a converted neuron co-stained with GFP, NeuN, and synaptophysin. Arrowheads indicate co-labeled cells. Scale bar, 50 µm. **C** Phase and fluorescent images of a converted neuron. Arrowheads indicated converted cells. Scale bar, 20 µm. **D** NeuroD1-converted neurons displayed repetitive action potential firing (60 dpi, *n* = 12). **E** Quantification of the frequency of action potential firing in cortical slices with ischemic injury (blue line, control; red line, NeuroD1 group, *n* = 5). Data are presented as means ± SEM. Note that the NeuroD1 group showed a significantly higher frequency of action potential firing than the control group

To assess synaptic connectivity, we stained for synaptophysin to visualize synaptic vesicles. Synaptophysin was observed in converted cells at 60 dpi (Fig. 6A, B), indicating the presence of synaptic structures. To further investigate whether NeuroD1-converted neurons have electrophysiological characteristics of functional excitable neurons, we measured the relationship between stimulating inputs and action potential (AP) firing frequency in NeuroD1-converted neurons using cortical slice whole-cell patch-clamp recordings in current clamp mode at 60 dpi. Cortical slice recordings were performed on GFP-labeled cells of the AAV-NeuroD1 and AAV-GFP groups (Fig. 6C–E). GFP^+^ cells in the AAV-NeuroD1 group exhibited repetitive APs, their firing frequency increased as higher amplitudes of current were applied, ranging from 50 to 350 pA (Fig. 6D). By contrast, cells in the AAV-GFP group showed little or no AP firing across the entire range of current amplitude (Fig. 6E).

### Astrocyte-to-neuron conversion attenuates microglial inflammatory responses and reduces glial scars after HIBI

**Fig. 7:**
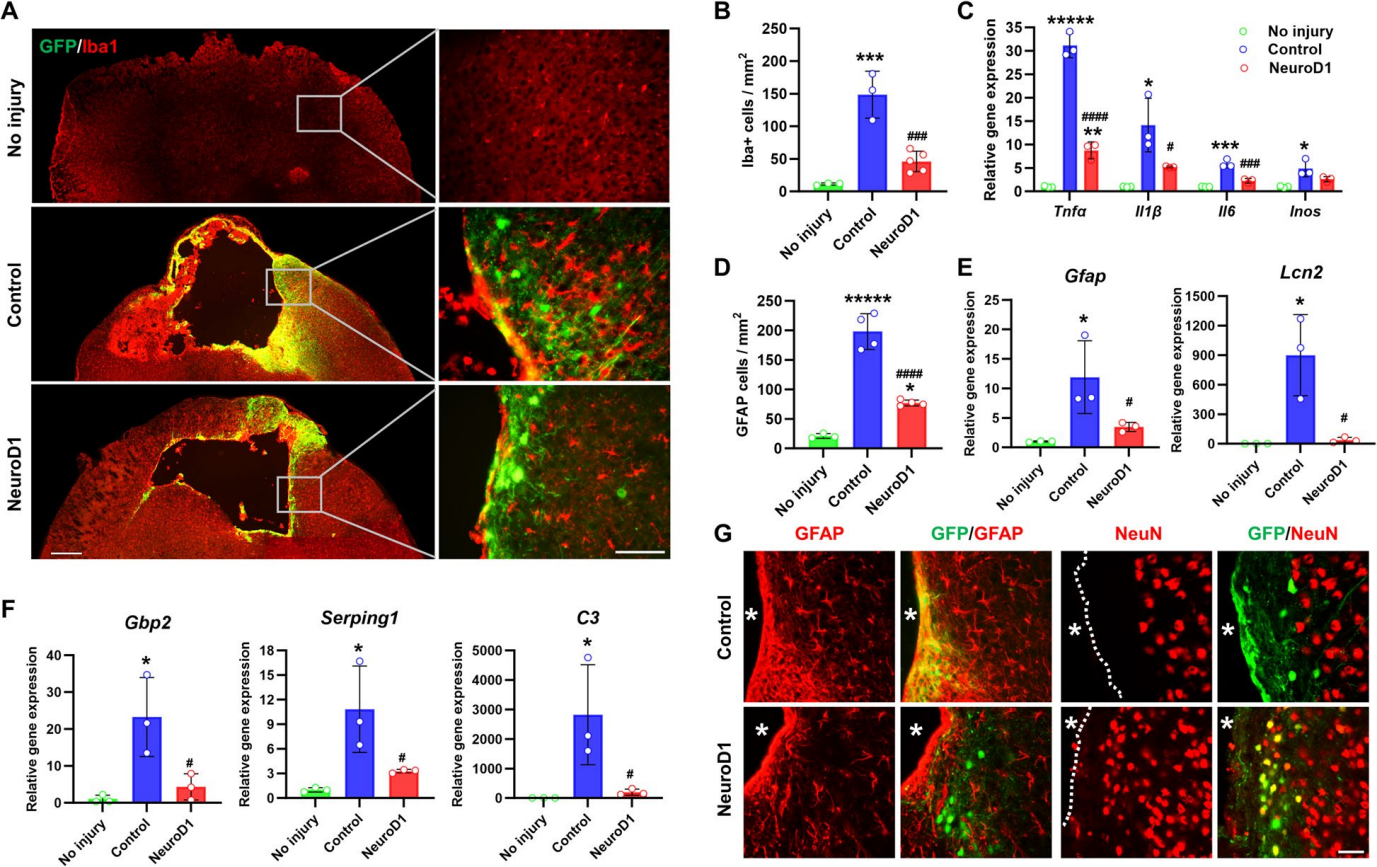
Astrocyte-to-neuron conversion by NeuroD1 attenuated microglial inflammatory responses and reduced glial scar after hypoxic-ischemic brain injury. **A** Low-magnification (left) and high-magnification (right) fluorescence images showing a decrease in microglial (Iba1, red) number in the ipsilateral hemisphere of the AAV-NeuroD1 group (NeuroD1, bottom) compared with the control AAV-GFP group (control, middle). Scale bar, 400 µm (left panels, low magnification); 100 µm (right panels, high magnification). **B** Quantitative analysis of the number of microglia around the injury sites (*n* = 3–5 mice per group). ***p < 0.001 vs no injury and ###*p* < 0.001 vs control. **C** Analysis of pro-inflammatory cytokine mRNA levels in hypoxic-ischemic-injured ipsilateral hemisphere at 4 weeks post-injection (*n* = 3–5 mice per group). *p < 0.05, **p < 0.01, ***p < 0.001, *****p < 0.00001 vs no injury and #*p* < 0.05, ###*p* < 0.001, ####*p* < 0.0001 vs control. Images in G showing accumulated astrocytes expressing GFAP (red). **D** Cell numbers were significantly decreased in the AAV-NeuroD1 group (*n* = 3 mice per group). *p < 0.05, *****p < 0.00001 vs no injury and ####*p* < 0.0001 vs control. **E**, **F** Analysis of relative expression of reactive astrocyte genes Gfap and Lcn2 and A1-type astrocyte-specific genes Gbp2, Serping1, and C3 (*n* = 3 mice per group). *p < 0.05 vs no injury and #*p* < 0.05 vs control. **G** Some newly converted neurons (GFP + and NeuN + cells) were observed in the peri-infarct area in which no neuronal cells are expected to survive after hypoxic-ischemic brain injury. An asterisk marks the infarct area. Dashed lines in g indicate the border of the infarct core. Scale bar, 50 µm. Data are presented as means ± SEM. One-way ANOVA followed by a Bonferroni post hoc test

As microglia activation contributes to inflammation in HIBI, we evaluated the effect of in vivo direct neuronal reprogramming on microglia at 30 dpi. Immunostaining with Iba1, a marker for microglia and macrophages, revealed a significant increase in AAV-GFP-infected areas after HIBI, whereas AAV-NeuroD1-infected areas exhibited a significant decrease (Fig. 7A, B). Microglia release pro-inflammatory cytokines (tumor necrosis factor alpha (TNFα), interleukin 1 beta (IL1β), interleukin 6 (IL6), and inducible nitric oxide synthase (Inos)), which cause neurotoxicity. qRT-PCR analysis showed increased cytokine expression post-HIBI; however, *Tnfα*, *Il1β*, and *Il6* were significantly downregulated in the AAV-NeuroD1 group. *Inos* expression was also weakened, but with no statistical significance, in the AAV-NeuroD1 group (Fig. 7C).

We also investigated the effect of in vivo direct neuronal reprogramming on reactive astrocytes that became active in response to HIBI. Immunostaining revealed decreased GFAP levels in the NeuroD1-treated peri-infarct area at 30 dpi (Fig. 7D, G). Some newly converted neurons (GFP^+^ and NeuN^+^) were observed in the peri-infarct area, which is typically devoid of neuronal cells following HIBI (Fig. 7G). qRT-PCR analysis of ipsilateral hemisphere tissue at 30 dpi revealed that the AAV-GFP group had a 12-fold increase in the pan-reactive astrocyte gene *Gfap* and an 800-fold increase in the reactive astrocyte-related neuroinflammatory marker *lipocalin 2* (*Lcn2*), whereas the AAV-NeuroD1 group exhibited significant decreases in *Gfap* and *Lcn2* expressions (Fig. 7E). Furthermore, the characteristic upregulation of A1-type reactive astrocyte genes (*Serping1*, *Gbp2*, and *C3*) after HIBI was significantly attenuated by NeuroD1 treatment (Fig. 7F). By contrast, the expression of the A2-type reactive astrocyte-specific gene *S100a10* did not change despite the decrease in GFAP expression after NeuroD1-mediated astrocyte-to-neuron conversion (Additional file 4: Fig. S4). These results indicate that NeuroD1-mediated in vivo direct neuronal reprogramming via AAVShH19 attenuated the reactive and neuroinflammatory properties of microglia and astrocytes.

### Astrocyte-to-neuron conversion improves function in HIBI

**Fig. 8:**
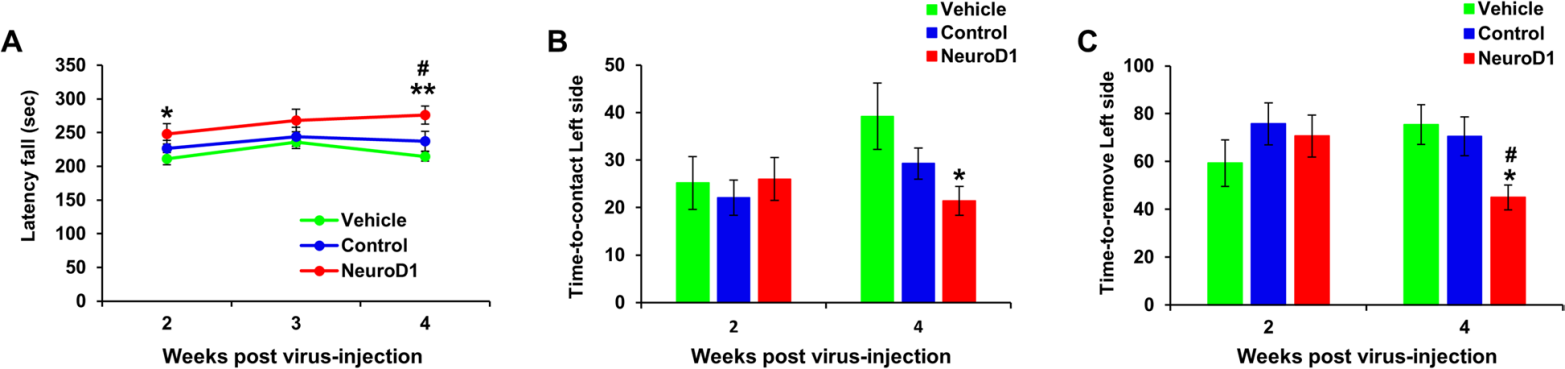
Neuronal conversion mediated by NeuroD1 improved functional outcomes after hypoxic-ischemic brain injury. **A**–**C** After injection of AAV-NeuroD1 (NeuroD1, *n* = 21); control AAV-GFP (control, *n* = 19); and vehicle (*n* = 18), rotarod (**A**) and adhesive tape removal (**B**, **C**) tests were performed for behavioral analysis. The rotarod test was performed 2, 3, and 4 weeks post-injection, and the adhesive tape removal test was performed 2 and 4 weeks post-injection. Data are presented as means ± SEM. *p < 0.05, **p < 0.001 vs vehicle and #*p* < 0.05 vs control. One-way ANOVA followed by a Bonferroni post hoc test

We evaluated whether NeuroD1-mediated astrocyte-to-neuron conversion could alleviate HIBI-induced neurobehavioral deficits. Rotarod and adhesive removal tests were conducted at various post-injection time points (Fig. 8). The results of the rotarod test did not differ between AAV-NeuroD1 and AAV-GFP groups at 2 weeks post-injection. However, the AAV-NeuroD1 group spent significantly more time on the rotarod than the vehicle group (248.0 ± 8.9 s vs. 211.4 ± 15.0 s; Fig. 8A). The performance of the AAV-NeuroD1 group improved over time and was significantly longer than that of the AAV-GFP group at 4 weeks post-injection (275.9 ± 7.1 s vs. 237.2 ± 14.7 s; Fig. 8A). Rotarod performance did not differ between the AAV-GFP and vehicle groups at any time point (Fig. 8a). The adhesive removal test, which assesses sensorimotor function, was conducted 2–4 weeks post-injection. On week 2, all groups showed similar performance. However, on week 4, the AAV-NeuroD1 group exhibited significantly faster contact (21.4 vs. 39.2 s; Fig. 8B) and removal times (45.0 vs. 75.5 s; Fig. 8C) compared to those of the vehicle group. The AAV-NeuroD1 group also had improved removal times compared with the AAV-GFP group (45.0 vs. 75.5 s; Fig. 8C). Taken together, NeuroD1-mediated astrocyte-to-neuron conversion can alleviate HIBI-induced neurobehavioral deficits.

## Discussion

In this study, direct neuronal reprogramming using AAV-mediated NeuroD1 gene delivery generated functional new neurons and improved neurobehavioral deficits in vivo in the mouse model of neonatal HIBI. Additionally, in vitro experiments demonstrated that ectopic NeuroD1 expression in astrocytes could efficiently convert astrocytes into neurons. Functional electrophysiological properties were observed in the newly converted neurons, which integrated with the preexisting host neurons. Furthermore, direct neuronal reprogramming with NeuroD1 protected preexisting neurons from HIBI and reduced toxic A1-type astrocyte gene expression, microglia activity, and neuroinflammation at the injury site. Therefore, in vivo NeuroD1-mediated direct neuronal reprogramming not only induced neuronal regeneration but also protected the microenvironment from further damage in the affected area (Additional file 5: Fig. S5). This overall effect led to a significant improvement in behavioral outcomes.

The direct conversion of glia into neurons has been a prominent research topic in recent years [4, 16]. Glia cells, abundant in the mammalian brain, have a close lineage to neurons and possess innate proliferative capacity, making them a promising candidate for neuronal reprogramming in the pursuit of neuroregeneration [17]. In the past few years, the direct in vivo conversion of reactive astrocytes into mature neurons has proven to be effective in both the adult mouse and non-human primate brain [4, 6–8, 18–21]. Additionally, strategies exist for direct in vivo conversion of microglia into mature neurons, and it has been shown that microglia in the striatum can be converted into striatal medium spiny neurons [22, 23]. In our study, we found that the virus infects predominantly specific cortical astrocytes in HIBI, and only a small amount of the virus infects striatal astrocytes, leading to their conversion into striatal medium spiny neurons. However, we confirmed that neurons converted from cortical astrocytes do not express markers of striatal medium spiny neurons, DARPP32 (data not shown). This suggests that the types of neurons generated by in vivo conversion systems may vary depending on the brain region and the characteristics of the target cells. In this study, we examined cortical astrocytes as the target cells for in vivo conversion strategy. Under normal conditions, astrocytes remain in a quiescent state and do not undergo activation or proliferation [24, 25], However, in response to brain injuries (e.g., brain ischemia, tumors, mechanical trauma, and neurodegenerative disorders), they become reactive, leading to altered gene expression and accelerated proliferation [14, 17, 25]. Reactive astrocytes are identified by increased GFAP expression [26]. To specifically target astrocytes in this study, we employed the GFAP promoter, Cre-FLEX system, and AAV technology. Due to its high infectivity, low pathogenicity, and intrinsic safety, AAV has emerged as a powerful gene delivery tool for treating various human diseases in the field of gene therapy [27, 28]. The AAV mutant ShH19 exhibited 5.5-fold higher astrocyte transduction than parental AAV2 in the rat striatum; however, only 15% of astrocytes were transduced in a previous study [12]. After confirming the distribution of a clear GFP signal in the peri-infarct area through the injection of AAVShH19 encoding GFP into HIBI, the efficiency of specific gene delivery to astrocytes was enhanced using the GFAP promoter (Additional file 6: Fig. S6). Our research data confirmed that hypoxic-ischemic brain injury in the neonatal period triggered the activation of astrocytes around the damaged brain tissue, and the injected NeuroD1 virus predominantly infected and expressed in astrocytes in cortical areas adjacent to the infarct site. The Cre-FLEX system enabled distinguishing between preexisting host neurons and transformed neurons, making it a suitable method for long-term monitoring of transformed neurons that originated from astrocytes [29].

NeuroD1 is a transcription factor that plays a crucial role in embryonic brain development and inducing neuronal differentiation. NeuroD1 induces neuronal fate decisions by directly binding to regulatory elements such as enhancers and promoters of genes involved in neuronal development [30–32]. Recent works have demonstrated the conversion of reactive astrocytes to glutamatergic neurons through NeuroD1 delivery in a middle cerebral artery occlusion model and Alzheimer’s disease model using lentiviruses and retroviruses, respectively [6, 33]. In a study on spinal cord injury, NeuroD1 overexpression using a retrovirus or AAV resulted in the transformation of astrocytes into neurons [34]. Furthermore, NeuroD1 expression through AAV9 reprogrammed astrocytes into both glutamatergic neurons and cortical neurons in a mouse model of stab injury [35]. The neuronal conversion of astrocytes was also observed in a rhesus monkey cerebral ischemia model through the delivery of AAV-carrying NeuroD1 [21]. Additionally, prior research has demonstrated that signals in vivo, e.g., different immune and inflammatory responses, growth factors, morphogens, and cytokines, can significantly affect different aspects of neuronal conversion [36]. Moreover, astrocytes have been observed to differentiate into neurons with different phenotypes via the same transcription factors, depending on the specific brain regions involved [37]. It has also been suggested that the intrinsic characteristics of astrocytes in various brain regions, combined with the local environmental niches, collectively play a role in shaping neuronal subtypes during in vivo direct reprogramming [38].

In this study, we conducted the first direct reprogramming investigation on a severe neonatal brain injury, HIBI. We observed that in neonatal mice with HIBI, NeuroD1 predominantly converted astrocytes into GABAergic neurons. This is significant because GABAergic neurons are known to be severely damaged by HIBI [39–41], which can decrease the expression of the GABA synthase enzymes GAD65 and GAD67 in the hippocampus after neonatal HIBI [42, 43]. The dysfunction and loss of GABAergic neurons can impair neural circuits and cognitive function [44]. Thus, the recovery of normal GABAergic neuron function in cerebral ischemia has been identified as a potential strategy to protect neurons and improve cognitive outcomes [44]. As the exact mechanism underlying the production of GABAergic neurons has not been fully elucidated in this study, further investigation through single-cell chromatic analysis is needed in further research. The converted neurons in the NeuroD1 group exhibited electrophysiological properties and expressed synaptophysin, suggesting that they established synaptic connections. These results suggest that NeuroD1-mediated conversion from astrocytes to functional neurons in vivo could replace lost neurons and improve brain function affected by HIBI.

In our study, NeuroD1 consistently converted astrocytes into GABAergic neurons both in healthy astrocytes (in vitro) and astrocytes within the peri-infarct area of hypoxic-ischemic brain injury (in vivo). These results contrast with previous studies using adult disease models of ischemic stroke or other brain injury, where cortical astrocytes were dominantly converted into glutamatergic neurons by NeuroD1 [45]. We hypothesize that delivery of NeuroD1 to cortical astrocytes during developmental stage directs their fate towards GABAergic neurons, while there are still limitations in elucidating the exact mechanism. Indeed, the density of GABAergic neurons in the cerebral cortex and white matter rapidly increases over the second half of gestation, peaking at term and then declining during the first six months of life, suggesting that the perinatal period is a critical time for the development of GABAergic system [46, 47]. Additionally, reprogramming with the vector AAVShH19 with the human GFAP promoter was attempted for the first time in our study, so the fate of reprogrammed cells may be differently triggered compared to previous research. Recently, Xi et al. reported that when NeuroD1 was delivered to Müller cells using two different serotypes of AAV (AAV7m8 and AAV9, with different GFAP promoters), the final converted cells were also different [48]. To figure out the exact mechanism underlying the production of GABAergic neurons has not been fully elucidated in this study, further investigation through single-cell chromatic analysis is needed in further research.

Brain disorders cause activation of astrocytes, i.e., reactive astrocytes, which subsequently undergo reactive astrogliosis and form a glial scar around the injury site. Reactive astrocytes also secrete growth inhibitory and inflammatory factors, e.g., TNFα, Lcn2, and proteoglycan proteins, which impede neural repair and regeneration. As brain injuries progress, the glial scar becomes a significant physical and chemical barrier that hinders tissue repair and neuronal regeneration. This barrier can persist for a long time and greatly impairs brain function recovery, particularly during the later stages of brain injuries [15, 49–51]. Recent studies have categorized reactive astrocytes based on their functions and gene expression patterns into A1-type and A2-type astrocytes. A1-type astrocytes have detrimental effects on central nervous system damage, such as neuronal cell death and neurotoxic cytokine secretion, whereas A2-type astrocytes secrete neuroprotective factors and promote neuronal survival [52, 53]. Our data shows that the number of reactive astrocytes and the expression levels of A1-type astrocyte-specific genes (Serping1, Gbp2, and C3) [54, 55] significantly increased after HIBI but were significantly decreased in the NeuroD1-treated group. This indicates that direct NeuroD1-mediated neuronal reprogramming can help suppress the detrimental effects of reactive astrocytes in HIBI. In addition to astrocytes, microglia also accumulate rapidly around the lesion in HIBI and increase the expression of pro-inflammatory factors and neurotoxic cytokines that inhibit axonal regeneration. This activation of microglia contributes to secondary damage to neurons and inhibits the recovery process [56, 57]. Our study found that the expression levels of pro-inflammatory cytokines and the activation of microglia in the damaged cortex were significantly reduced in the AAV-NeuroD1-injected group compared with the AAV-GFP-injected group (Fig. 7A–C). Although it was not clear which cellular response occurred first, it is possible that the decreased number of reactive astrocytes and decreased microglia activation are related.

Previous studies have shown that neurotoxic reactive astrocytes can be induced by cytokines (e.g., IL1a, TNFα, and component 1 subcomponent q) that are released by reactive microglia [53, 58]. Astrocytes can also regulate microglial activity through various factors, such as Ca^2+^, ATP, chemokines, complement (C3), and plasma protein orosomucoid 2 [59–63]. By promoting direct astrocyte-to-neuron conversion, NeuroD1 indirectly modulates the reactivity of microglia and astrocytes at the site, leading to a reduction in neuroinflammation. This reduction in neuroinflammation improves the histological and functional outcomes of brain disease [54, 64–66]. Therefore, the protection of preexisting host neurons around the damaged area after NeuroD1-mediated neuron conversion observed in this study may be affected by microenvironmental changes, such as decreased numbers of reactive astrocytes and microglia, downregulated A1-type astrocyte genes, and decreased levels of pro-inflammatory factors. Direct NeuroD1-mediated neuronal reprogramming in vivo resulted in these effects, which demonstrated improvements in neurobehavioral function in an animal model of neonatal HIBI. These findings suggest that direct neuronal reprogramming may have therapeutic potential for treating brain injuries and diseases by promoting neural repair and regeneration while also modulating the immune response to injury. However, additional studies are required to fully understand the underlying mechanisms that produce these effects and to determine the clinical applicability of direct neuronal reprogramming in the treatment of brain diseases.

## Conclusion

To summarize, NeuroD1-mediated in vivo neuronal conversion via AAVShH19 efficiently converted astrocytes to neurons with functional electrophysiological properties, providing a potential strategy for replacing lost neurons caused by neonatal HIBI. Moreover, this approach reduced neuroinflammation and protected preexisting neurons, thereby mitigating sensorimotor deficits. Our findings suggest that in vivo neural regeneration via AAV-NeuroD1 gene delivery to astrocytes may represent a therapeutic strategy for neuronal regeneration in cases of neonatal HIBI.

### Supplementary Information

Additional file 1: Figure S1. Schematic illustration of gene cassettes encapsidated into AAV vectors.

Additional file 2: Figure S2. Schematic illustration of the Cre-FLEX system.

Additional file 3: Figure S3. NeuroD1 converted astrocytes into GABAergic neurons in the hypoxic-ischemic-injured brain. A, B A high-magnification confocal image and the three-dimensional reconstruction of the image showing expression of the GABAergic neuronal marker VGAT (red) in GFP + cells (green; overlay color: yellow). Scale bar, 50 μm. Arrowheads indicate co-labeled cells. C Quantitative analysis of GFP + cells co-labeled with VGAT. Note that 48.6% of converted cells express VGAT (*n *= 3 mice per group). Data are presented as means ± SEM. ***p < 0.001 vs control.

Additional file 4: Figure S4. Analysis for relative expression of A2-type astrocyte specific genes *S100a10* (*n* = 3 mice per group). Data are presented as means ± SEM.

Additional file 5: Figure S5. A schematic representation of therapeutic mechanisms of AAV-NeuroD1 gene delivery into neonatal hypoxic-ischemic brain injury.

Additional file 6: Figure S6. A Low-magnification fluorescence image showing GFP and GFAP signal in the peri-infarct cortex in the ipsilateral hemisphere after injection of AAV GFAP::Cre together with AAV CAG::FLEX-NeuroD1-T2A-GFP (AAV-NeuroD1) into HIBI. Scale bar, 500 μm. B AAV-NeuroD1 efficiently infected reactive astrocytes in cortical areas adjacent to the infarct site (high-magnification). Scale bars, 200 μm (upper panel). Scale bars, 50 μm. (bottom panel).

## Data Availability

The datasets used and/or analyzed during the current study are available from the corresponding author on reasonable request.

## Abbreviations

HI: Hypoxic-ischemic
NeuroD1: Neurogenic differentiation factor 1
AAV: Tropic adeno-associated virus
FBS: Fetal bovine serum
PBS: Phosphate-buffered saline
DMEM: Dulbecco’s modified Eagle’s Medium
BSA: Bovine serum albumin
NDS: Normal donkey serum
GFAP: Glial fibrillary acidic protein
S100β: S100 calcium-binding protein beta
NeuN: Neuronal nuclei
TUJ1: Neuronal class III β-tubulin
Iba-1: Anti-ionized calcium-binding adapter molecule 1
ChAT: Choline acetyltransferase
GAD67: Glutamic acid decarboxylase 67
GABA: Gamma-aminobutyris acid
VGluT1: Vesicular glutamate transporter 1
Olig2: Oligodendrocyte transcription factor 2
DAPI: 40,6-Diamidino-2-phenylindole
OCT: Optimal cutting temperature
DCX: Doublecortin
GS: Glutamine synthetase
Lcn2: Lipocalin 2
C3: Complement
