# Supplementary figures and images for: In vivo neural regeneration via AAV-NeuroD1 gene delivery to astrocytes in neonatal hypoxic-ischemic brain injury
Source: Inflamm Regen. 2024 Jul 16;44:33. doi: 10.1186/s41232-024-00349-y (PMC11253351; doi:10.1186/s41232-024-00349-y)

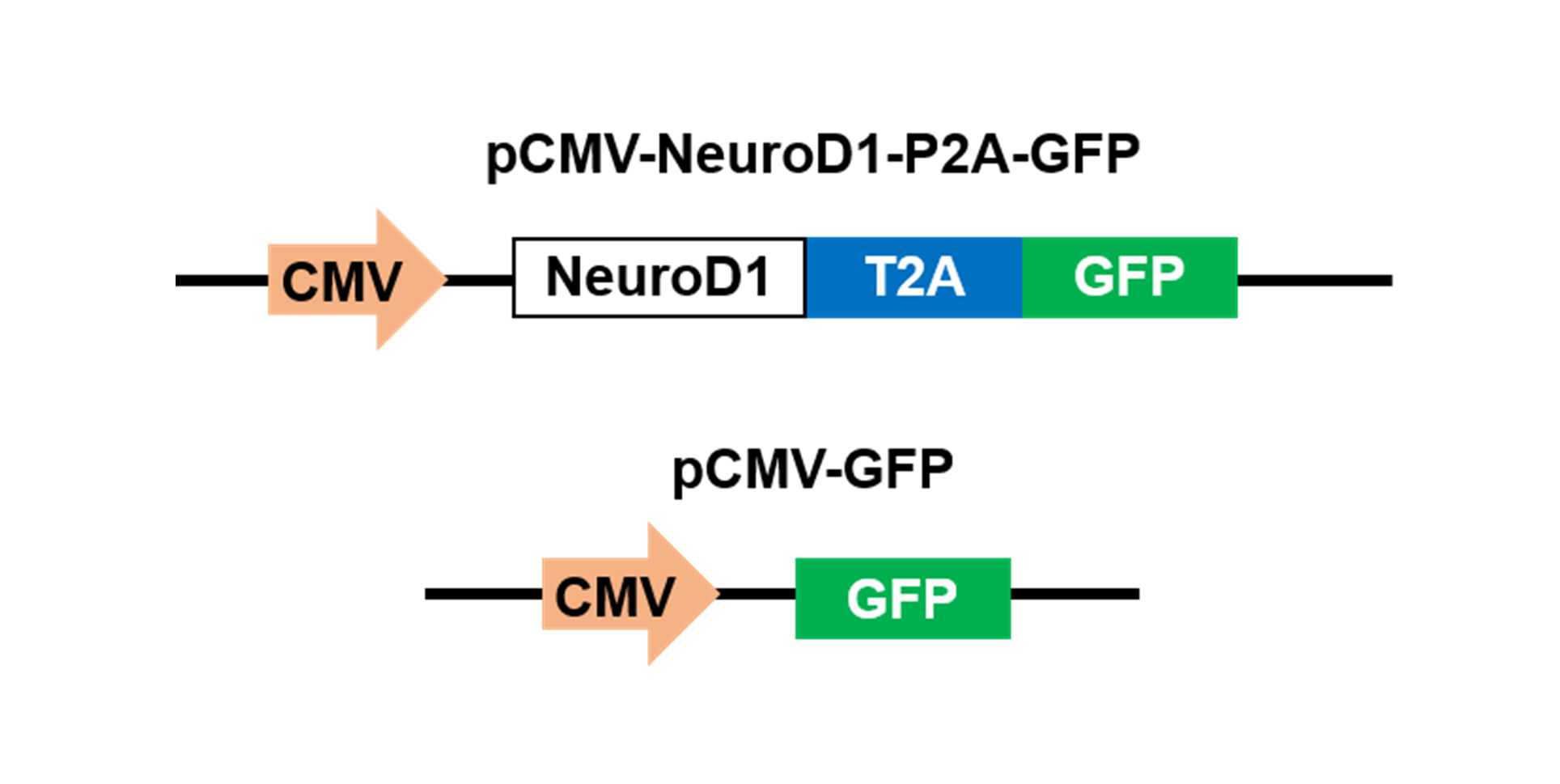

Supplement: Supplementary file 1 — Additional file 1: Figure S1. Schematic illustration of gene cassettes encapsidated into AAV vectors. [file 41232_2024_349_MOESM1_ESM.tif]

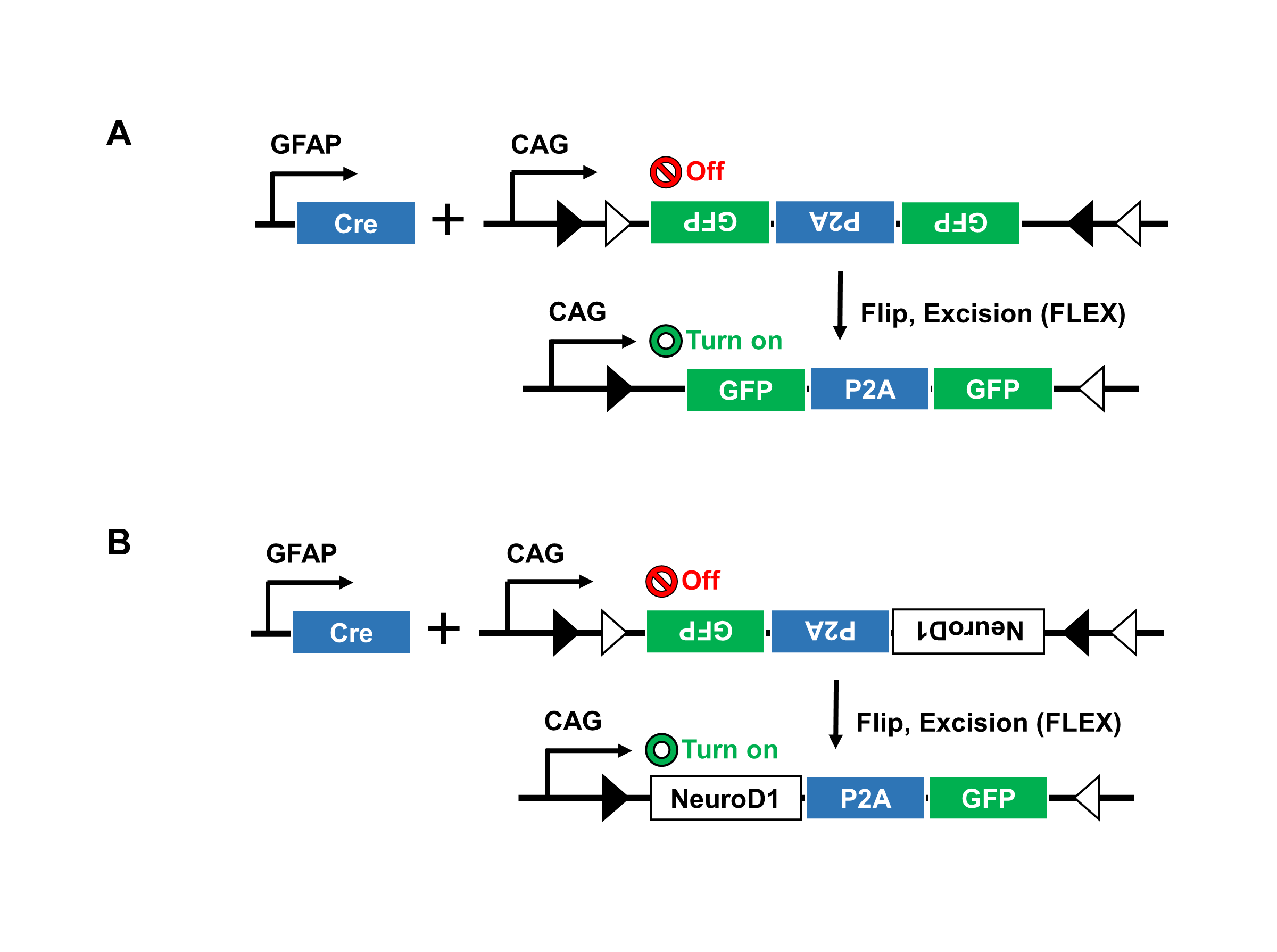

Supplement: Supplementary file 2 — Additional file 2: Figure S2. Schematic illustration of the Cre-FLEX system. [file 41232_2024_349_MOESM2_ESM.tif]

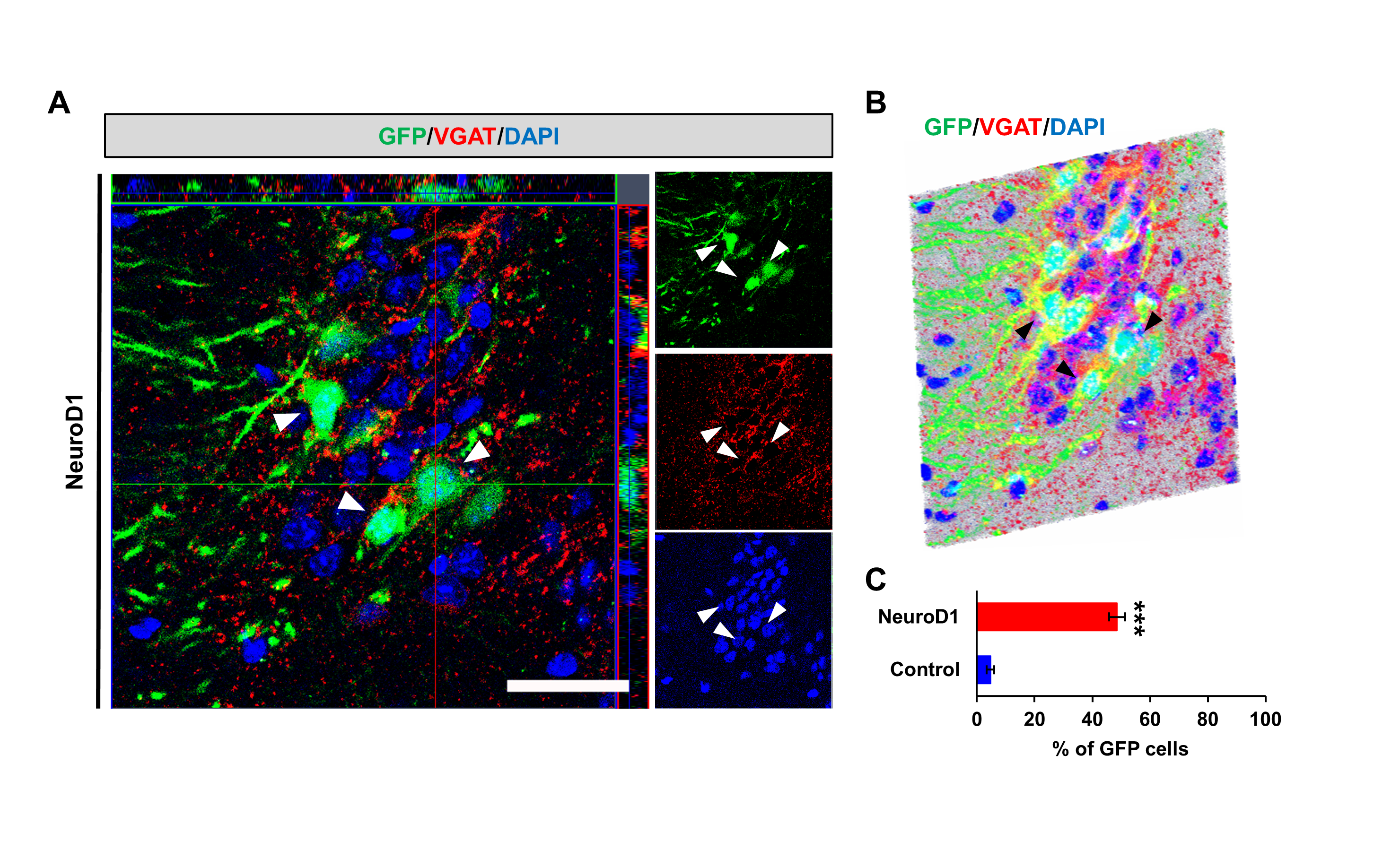

Supplement: Supplementary file 3 — Additional file 3: Figure S3. NeuroD1 converted astrocytes into GABAergic neurons in the hypoxic-ischemic-injured brain. A, B A high-magnification confocal image and the three-dimensional reconstruction of the image showing expression of the GABAergic neuronal marker VGAT (red) in GFP + cells (green; overlay color: yellow). Scale bar, 50 μm. Arrowheads indicate co-labeled cells. C Quantitative analysis of GFP + cells co-labeled with VGAT. Note that 48.6% of converted cells express VGAT (n = 3 mice per group). Data are presented as means ± SEM. ***p < 0.001 vs control. [file 41232_2024_349_MOESM3_ESM.tif]

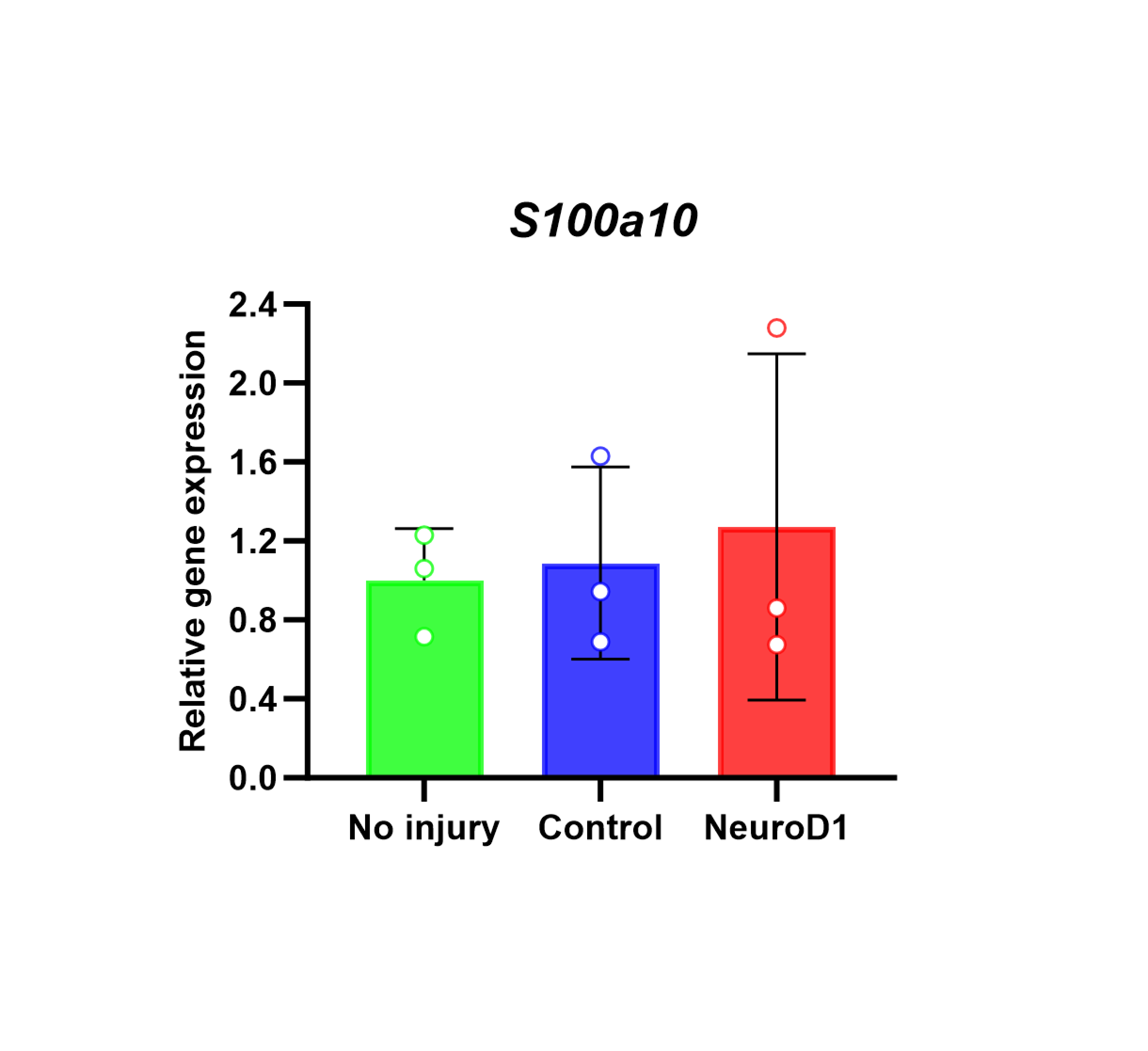

Supplement: Supplementary file 4 — Additional file 4: Figure S4. Analysis for relative expression of A2-type astrocyte specific genes S100a10 (n = 3 mice per group). Data are presented as means ± SEM. [file 41232_2024_349_MOESM4_ESM.tif]

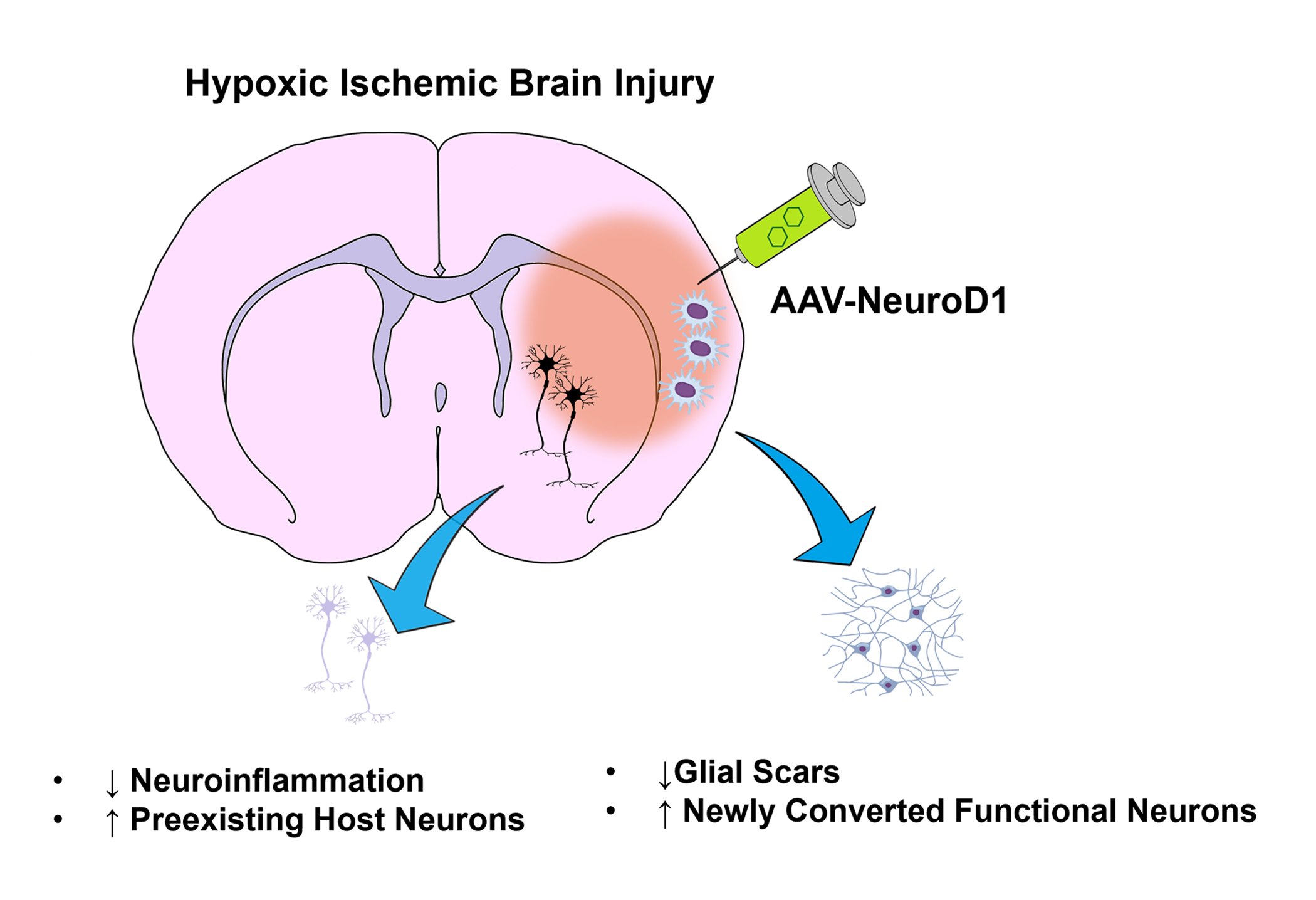

Supplement: Supplementary file 5 — Additional file 5: Figure S5. A schematic representation of therapeutic mechanisms of AAV-NeuroD1 gene delivery into neonatal hypoxic-ischemic brain injury. [file 41232_2024_349_MOESM5_ESM.tif]

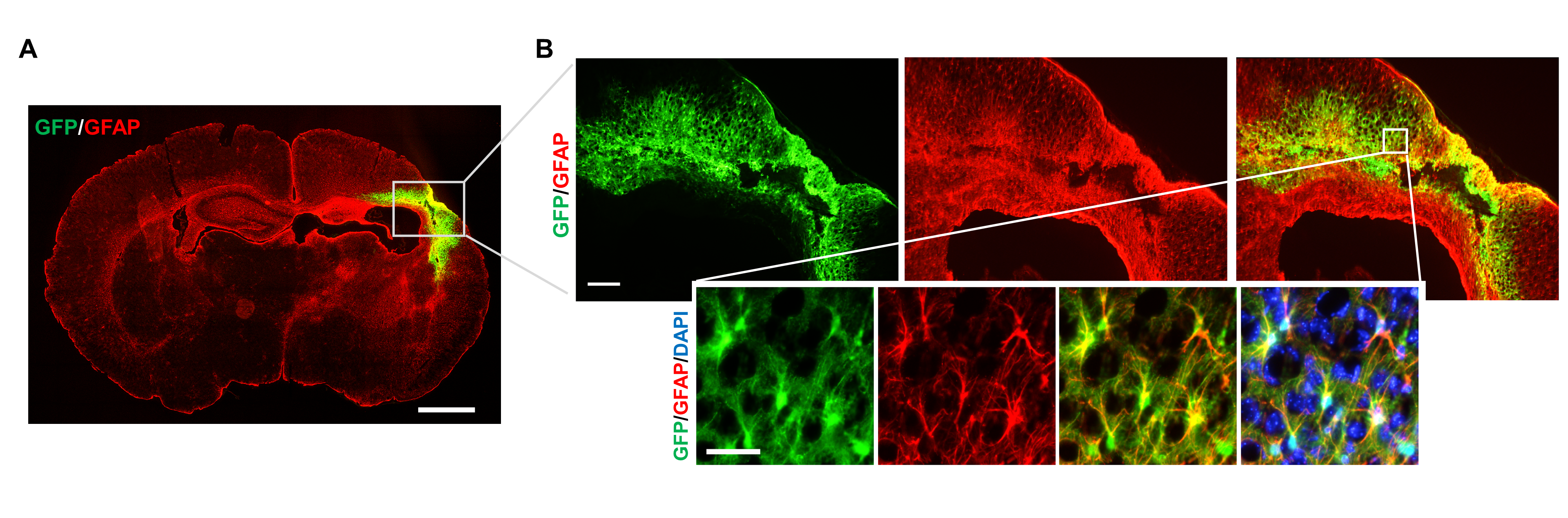

Supplement: Supplementary file 6 — Additional file 6: Figure S6. A Low-magnification fluorescence image showing GFP and GFAP signal in the peri-infarct cortex in the ipsilateral hemisphere after injection of AAV GFAP::Cre together with AAV CAG::FLEX-NeuroD1-T2A-GFP (AAV-NeuroD1) into HIBI. Scale bar, 500 μm. B AAV-NeuroD1 efficiently infected reactive astrocytes in cortical areas adjacent to the infarct site (high-magnification). Scale bars, 200 μm (upper panel). Scale bars, 50 μm. (bottom panel). [file 41232_2024_349_MOESM6_ESM.tif]
